# Supplementary material for: The Healthy Brain Network Serial Scanning Initiative: a resource for evaluating inter-individual differences and their reliabilities across scan conditions and sessions
Source: Gigascience. 2017 Jan 7;6(2):1–14. doi: 10.1093/gigascience/giw011 (PMC5466711; doi:10.1093/gigascience/giw011)
Supplement: GIGA-D-16-00112_Revision_2.pdf [file giw011_GIGA-D-16-00112_Revision_2.pdf]

GigaScience

The Healthy Brain Network Serial Scanning Initiative: A resource for evaluating inter-individual differences and their reliabilities across scan conditions and sessions

--Manuscript Draft--

Manuscript Number:	GIGA-D-16-00112R2
Full Title:	The Healthy Brain Network Serial Scanning Initiative: A resource for evaluating inter-individual differences and their reliabilities across scan conditions and sessions
Article Type:	Data Note
Funding Information:	Lee Alexander, Robert Allard, Lisa Bilotti Foundation, Inc., Margaret Billoti, Christopher Boles, Brooklyn Nets, Agapi and Bruce Burkhard, Randolph Cowen and Phyllis Green, Elizabeth and David DePaolo, Charlotte Ford, Valesca Guerrand-Hermes, Sarah and Geoffrey Gund, George Hall, Joseph Healey and Elaine Thomas, Hearst Foundations, Eve and Ross Joffe, Anton and Robin Katz, Rachael and Marshall Levine, Ke Li, Jessica Lupovici, Javier Macaya, Christine and Richard Mack, Susan Miller and Byron Grote, John and Amy Phelan, Linnea and George Roberts, Jim and Linda Robinson Foundation, Inc, Caren and Barry Roseman, Zibby Schwarzman, David Shapiro and Abby Pogrebin, Stavros Niarchos Foundation, Nicholas Van Dusen, David Wolkoff and Stephanie Winston Wolkoff and the Donors to the Brant Art Auction of 2012. (N/A) — Dr. Michael Peter Milham
Abstract:	Background<br>Although typically measured during the resting state, a growing literature is illustrating the ability to map intrinsic connectivity in task and naturalistic viewing fMRI paradigms. These paradigms are drawing excitement due to their greater tolerability in clinical and developing populations and because they enable a wider range of analyses (e.g. inter-subject correlations). To be clinically useful, the test-retest reliability of connectivity measured during these paradigms needs to be established. This resource provides data for evaluating test-retest reliability for full-brain connectivity patterns detected during each of four scan conditions that differ with respect to level of engagement (rest, abstract animations, movie clips, flanker task). Data is provided for thirteen participants, each scanned in twelve sessions with 10 minutes for each scan of the four conditions. Diffusion kurtosis imaging data was also obtained at each session.<br><br>Findings<br>Technical validation and demonstrative reliability analyses were carried out at the connection-level using the Intraclass Correlation Coefficient (ICC), and at network level representations of the data using the Image Intraclass Correlation Coefficient (I2C2). Variation in intrinsic functional connectivity across sessions was generally found to be greater than that attributable to scan condition. Between-condition reliability was generally high, particularly for the frontoparietal and default networks. Between-session reliabilities obtained separately for the different scan conditions were comparable, though notably lower than between-condition reliabilities.<br><br>Conclusions<br>The described resource provides a test-bed for quantifying the reliability of connectivity indices across conditions and time. The resource can be used to compare and optimize different frameworks for measuring connectivity and data collection parameters such as scan length. Additionally, investigators can explore the unique perspectives of the brain's functional architecture offered by each of the scan conditions.
Corresponding Author:	Michael Peter Milham, M.D., Ph.D.

Powered by Editorial Manager® and ProduXion Manager® from Aries Systems Corporation

	UNITED STATES
**Corresponding Author Secondary Information:**	
**Corresponding Author's Institution:**	
**Corresponding Author's Secondary Institution:**	
**First Author:**	David O'Connor
**First Author Secondary Information:**	
**Order of Authors:**	David O'Connor
	Natan Vega Potler, B.A.
	Meagan Kovacs, M.S.
	Ting Xu, Ph.D.
	Lei Ai, Ph.D.
	John Pellman, B.A.
	Tamara Vanderwal, M.D.
	Lucas Parra, Ph.D.
	Samantha Cohen, M.A.
	Satrajit Ghosh, Ph.D.
	Jasmine Escalera, Ph.D.
	Natalie Grant-Villegas, B.A.
	Yael Osman, B.A.
	Anastasia Bui, B.A.
	Richard Cameron Craddock, Ph.D.
	Michael Peter Milham, M.D., Ph.D.
**Order of Authors Secondary Information:**	
**Response to Reviewers:**	Updated manuscript with GigaScience DOI, and correct face masking software.<br><br>Updated Ref 71 with DOI citation details.
**Additional Information:**	
**Question**	**Response**
Are you submitting this manuscript to a special series or article collection?	No
**Experimental design and statistics**<br><br>Full details of the experimental design and statistical methods used should be given in the Methods section, as detailed in our Minimum Standards Reporting Checklist. Information essential to interpreting the data presented should be made available in the figure legends.<br><br>Have you included all the information requested in your manuscript?	Yes

**Resources**<br><br>A description of all resources used, including antibodies, cell lines, animals and software tools, with enough information to allow them to be uniquely identified, should be included in the Methods section. Authors are strongly encouraged to cite Research Resource Identifiers (RRIDs) for antibodies, model organisms and tools, where possible.<br><br>Have you included the information requested as detailed in our Minimum Standards Reporting Checklist?	Yes
**Availability of data and materials**<br><br>All datasets and code on which the conclusions of the paper rely must be either included in your submission or deposited in publicly available repositories (where available and ethically appropriate), referencing such data using a unique identifier in the references and in the "Availability of Data and Materials" section of your manuscript.<br><br>Have you have met the above requirement as detailed in our Minimum Standards Reporting Checklist?	Yes

Manuscript
Click here to download Manuscript hbnss_manuscript_finaldraft_postreview_final 2.docx

RUNNING HEAD: Serial Scanning Initiative

The Healthy Brain Network Serial Scanning Initiative: A resource for evaluating inter-individual differences and their reliabilities across scan conditions and sessions

David O'Connor[1,2], Natan Vega Potler[1], Meagan Kovacs[1],Ting Xu[1], Lei Ai[1], John Pellman[1,2], Tamara Vanderwal[3], Lucas Parra[4], Samantha Cohen[5], Satrajit Ghosh[6], Jasmine Escalera[1], Natalie Grant-Villegas[1], Yael Osman[1], Anastasia Bui[1], R. Cameron Craddock[1,2], Michael P. Milham[1,2]*

1. Child Mind Institute Healthy Brain Network, New York, New York
2. Center for Biomedical Imaging and Neuromodulation, Nathan S. Kline Institute for Psychiatric Research, Orangeburg, New York
3. Yale University, New Haven, Connecticut
4. City College of New York, New York, New York
5. The Graduate Center of the City University of New York, New York, New York
6. Massachusetts Institute of Technology, Cambridge, Massachusetts

*Correspondence:

Michael Peter Milham, MD, PhD

Center for Developing Brain

Child Mind Institute

New York, NY 10022, USA

Michael.Milham@childmind.org (M.P. Milham)

Abstract

Background

Although typically measured during the resting state, a growing literature is illustrating the ability to map intrinsic connectivity in task and naturalistic viewing fMRI paradigms. These paradigms are drawing excitement due to their greater tolerability in clinical and developing populations and because they enable a wider range of analyses (e.g. inter-subject correlations). To be clinically useful, the test-retest reliability of connectivity measured during these paradigms needs to be established. This resource provides data for evaluating test-retest reliability for full-brain connectivity patterns detected during each of four scan conditions that differ with respect to level of engagement (rest, abstract animations, movie clips, flanker task). Data is provided for thirteen participants, each scanned in twelve sessions with 10 minutes for each scan of the four conditions. Diffusion kurtosis imaging data was also obtained at each session.

Findings

Technical validation and demonstrative reliability analyses were carried out at the connection-level using the Intraclass Correlation Coefficient (ICC), and at network level representations of the data using the Image Intraclass Correlation Coefficient (I2C2). Variation in intrinsic functional connectivity across sessions was generally found to be greater than that attributable to scan condition. Between-condition reliability was generally high, particularly for the frontoparietal and default networks. Between-session reliabilities

obtained separately for the different scan conditions were comparable, though notably lower than between-condition reliabilities.

Conclusions

The described resource provides a test-bed for quantifying the reliability of connectivity indices across conditions and time. The resource can be used to compare and optimize different frameworks for measuring connectivity and data collection parameters such as scan length. Additionally, investigators can explore the unique perspectives of the brain's functional architecture offered by each of the scan conditions.

Keywords

fMRI, Data Sharing, Reliability

DATA NOTE

Data Description

An extensive literature has documented the utility of fMRI for mapping the brain's functional interactions through the detection of temporally correlated patterns of spontaneous activity between spatially distinct brain areas [1]–[7]. Commonly referred to as intrinsic functional connectivity (iFC), these patterns are commonly studied during the 'resting state', which involves the participant quietly lying awake and not performing an externally driven task. Resting state fMRI (R-fMRI) has gained popularity in clinical neuroimaging due to its minimal task and participant compliance demands. R-fMRI has also demonstrated good test-retest reliability for commonly used measures [8]–[12], and utility in detecting brain differences associated with neuropsychiatric disorders [13], [14]. Despite these successes, a growing body of work is questioning the advantages of resting state, given reports of higher head

motion, decreased tolerance of the scan environment (e.g. boredom, rumination), and increased likelihood of falling asleep compared to more engaging task-based fMRI paradigms [15]–[18]. This is particularly relevant for studies of pediatric, geriatric and clinical populations, all of which are characterized by lower tolerance of the scanner environment.

A number of less challenging scan conditions have been proposed as alternatives for estimating iFC. Particularly intriguing are “naturalistic viewing” paradigms [15], [19], [20]. It has been shown that the mental state (i.e., emotional state, performing a task, etc.) of the participant during scanning can affect iFC patterns; recent work suggests that low engagement states (e.g., computer animations with limited cognitive content) may come close to mimicking rest from a neural perspective [21]. Several studies have illustrated the ability to relate trait phenotypic variables to inter-individual differences in iFC across conditions, even if extrinsically driven signals (i.e., task stimulus functions) are not removed [21]–[27]. However, comprehensive comparisons of the relative impact of scan condition on detection of inter-individual differences in intrinsic functional connectivity, and the test-retest reliability of these differences, are needed before these paradigms can fully supplant R-fMRI.

Here we describe a dataset that was generated as part of a pilot testing effort for the Child Mind Institute Healthy Brain Network – a large-scale data collection effort focused on the generation of an open resource for studying child and adolescent mental health. The primary goal of the data collection was to assess and compare test-retest reliability of full-brain connectivity patterns detected for each of four scan conditions that differed with respect to level of engagement. Specifically, 13 participants were scanned during each of the following four conditions on 12 different occasions: 1) rest, 2) free viewing of computer-generated abstract shapes with music designed to have minimal cognitive or emotional

content (i.e., "Inscapes", [15]), 3) free viewing of highly engaging movies [19], and 4) performance of an active task (i.e., an Erickson flanker task [28], with no-Go trials included). For each of the non-rest conditions, three different stimuli were used, with each being repeated four times across the 12 sessions to enable the evaluation of repetition effects. Given the focus on naturalistic viewing, an additional scan session containing a full viewing of “Raiders of the Lost Ark” (Lucasfilm Ltd., 1981) was included to facilitate interested parties in the exploration and evaluation of hyper alignment approaches, which offer increasingly popular and unique solutions to overcoming anatomical variability when attempting to match functional systems across individuals [29].

Although not a primary focus of the data collection, additional structural imaging data was collected, which are being shared as well: 1) MPRAGE [30], 2) diffusion kurtosis imaging [31], [32], 3) quantitative T1/T2 anatomical imaging (single session) [33], 4) magnetization transfer (single session) [34] (see Table 1). Functional MRI data from a single movie viewing session during which Raiders of the Lost Ark was viewed in its entirety, is included as well.

Table 1 – HBN-SSI experimental design.

Shared Imaging Data		
Session #	Session Type	Description
1	Baseline Characterization	• Multiecho MPRAGE<br>• Diffusion Kurtosis Imaging<br>• Quantitative T1/T2 Mapping<br>• Magnetization Transfer Ratio<br>• FLAIR<br>• fMRI: rest (10 min)
2-7, 9-14	Repeat Scanning	• Multiecho MPRAGE<br>• Diffusion Kurtosis Imaging<br>• fMRI: rest (10 min)<br>• fMRI: Naturalistic Viewing: Inscapes (10 min)<br>• fMRI: Naturalistic Viewing: Movie Clips (10 min)<br>• fMRI: Flanker Task (10 min)
8	Full Feature Movie	• fMRI: Raiders of the Lost Ark (20 min X 6)

METHODS

Participants and Procedures

13 adults (ages 18-45 years; mean age: 30.3; 38.4% male) recruited from the community participated in the Healthy Brain Network's Serial Scanning Initiative. Each participant attended 14 sessions over a period of 1-2 months; see Table 1 for the breakdown of data acquired across sessions. All imaging data were collected using a 1.5T Siemens Avanto equipped with a 32-channel head coil in a mobile trailer (Medical Coaches, Oneonta, NY). The scanner was selected as part of a pilot initiative being carried out to evaluate the capabilities of a 1.5T mobile scanner when equipped with a state-of-the-art head coil and imaging sequences. All research performed was approved by the Chesapeake Institutional Review Board, Columbia, MD [35].

Experimental Design. As outlined in Table 1, each participant attended a total of 14 separate imaging session; these included: 1) a baseline characterization session containing a variety of quantitative anatomical scans, 2) 12 serial scanning sessions, each using the same imaging protocol consisting of four functional MRI scan conditions (10 minutes per condition), diffusion kurtosis imaging and a reference MPRAGE anatomical scan, and 3) a 'Raiders of the Lost Ark' movie viewing session.

Functional MRI Scan Conditions Included in Serial Scanning. The following four functional scan conditions were selected to sample a range of levels of engagement, presented **in ascending order of level of engagement (See Figure 1):**

Rest

The participant was presented a white fixation cross in the center of a black screen and instructed to rest with eyes open. Specific instructions were as follows: “Please lie quietly with your eyes open, and direct your gaze towards the plus symbol. During this scan let your mind wander. If you notice yourself focusing on a particular stream of thoughts, let your mind wander away.”

Inscapes

Inscape is a computer generated animation comprised of abstract, non-social, technological-looking 3D forms that transition in a slow, continuous fashion without scene cuts. Visual stimulation is accompanied by a piano composition based on the pentatonic scale with a slow tempo (48 bpm), which was intended to be calming and to harmonize with the noise generated by EPI sequences [15]. Three unique 10 minute sequences were created using the original 7-minute Inscapes, and were presented across the 12 repeat scanning sessions. These clips are available for download from the HBN-SSI webpage [36].

Movie

Three unique 10-minute movie clips were presented across the 12 repeat scanning sessions. To ensure a high level of engagement, three Hollywood movie clips (American versions) were selected, each representing a different movie genre and containing a narrative arc that fit into the 10-minute clip. The specific clips selected were: Wall-E (Walt Disney Productions, 2008, time codes 00:02:03:13 to 00:12:11:05), The Matrix (Warner Bros., 1999, 00:25:23:10 to 00:35:19:20), and A Few Good Men (Columbia Pictures,1992, 01:58:13:01 to 02:08:11:18). Due to copyright issues these clips could not be shared.

Flanker

The Eriksen Flanker task consisted of presenting a series of images containing 5 arrows. For each image, the participant was asked to focus on the center arrow and indicate if it is pointing left or right by pushing a button with their left or right index finger. The flanking arrows could be pointing the same way (congruent) or the opposite way (incongruent). Also built into the task were a neutral stimulus and a go/no-go aspect. The neutral task would contain diamonds instead of flanking arrows, making the central arrow direction more obvious. The no-go stimuli contains x's instead of flanking arrows, indicating that the subject should not push either button. See Figure 1 for a visualization of the stimuli. The stimuli and timing of their presentation are available for download from the HBN-SSI webpage [36].

Counter-Balancing

Order effects are an obvious concern when comparing four functional scan conditions. To minimize these effects, we ensured that for each participant; 1) each scan type occurred an equal number of times in each of the four scan slots across the 12 sessions, and that 2) each scan type had an equal frequency of being preceded by each of the other three scan types. We made use of 3 exemplars of each non-rest stimuli to enable the examination of repetition effects. For movies, this involved having three 10-minute clips, each from a different movie; for Inscapes, this involved three different animation sequences and for the flanker task, three different stimulus orderings were used. We guaranteed that across the 12 scan sessions, each exemplar occurred one time across every three scan sessions. Specific ordering of exemplars were varied across 'odd' and 'even' numbered participants. For each participant, individual-specific ordering information is provided in the release.

Imaging Protocols (See Table 2 for scan protocol details).

- Functional MRI (sessions 1-14): For all functional MRI scans, the multiband EPI sequence provided by CMRR [37] was employed to provide high spatial and temporal resolutions (multiband factor 3, voxel size: 2.46x2.46x2.5mm; TR: 1.46 seconds).
- MEMPRAGE (sessions 1-7, 9-14): Across all sessions (except the full-movie session), we obtained a multi-echo MPRAGE sequence for the purposes of anatomical registration [38]. Within a given scan, four echoes are collected per excitation and combined using root mean square average. This enables the images to be acquired with a higher bandwidth to reduce distortion, while recovering SNR through averaging. The added T2* weighting from the later echoes also helps differentiate dura from brain - matter.
- Diffusional Kurtosis Imaging (DKI): Leveraging the capabilities of the CMRR multiband imaging sequence, we were able to acquire 64 directions at 2 b-values (1000 and 2000 s/mm^2). This enables diffusion kurtosis specific metrics to be calculated from the data in addition to standard DTI metrics and can improve tractography [31].
- Quantitative Relaxometry MRI (Quantitative T1, T2, and Myelin Water Fraction [MWF]): DESPOT1 and DESPOT2 sequences were used to characterize microstructural properties of brain tissue. These innovative acquisition strategies enable quantitation of T1 and T2 relaxation constants, which can be combined to calculate myelin water fraction [39].
- Magnetization Transfer: High-resolution T1-weighted structural images were acquired with a FLASH sequence, with and without a saturation RF pulse. The magnetization transfer ratio is calculated from the resulting images, which is purportedly a sensitive marker of myelination [34].

[Table 2 – MRI acquisition parameters for scans included in the HBN-SSI.]

Limitations

A limitation of the described resource is that the data was collected using a 1.5T scanner platform, rather than 3T. While we do not expect the overall results obtained with data from the 1.5T and 3T platforms should be fundamentally different, there is generally better SNR and temporal resolution with the 3T scanner platform. To mitigate these differences, 1) the system was upgraded to 32 receive channels to take advantage of the latest head-coil technologies for increasing SNR, and 2) simultaneous multi-slice imaging was used to improve the spatial and temporal resolution.

DATA RECORDS

Data Privacy

The HBN-SSI data are being shared via the 1000 Functional Connectomes Project and its International Neuroimaging Data-sharing Initiative (FCP/INDI) [40]. Prior to sharing, all imaging data were fully de-identified by removing all personally identifying information (as defined by the Health Insurance Portability and Accountability) from the data files, including facial features. The removal of facial features as performed using the “mri_deface” software package developed by Bischoff-Grethe et al. [41]. All data were visually inspected before release to insure that these procedures worked as expected.

Distribution for use

<u>Imaging Data</u>

All MRI data can be accessed through the Neuroimaging Informatics Tools and Resources Clearinghouse (NITRC) [36] and FCP/INDI’s Amazon Web Services public Simple Storage Service (S3) bucket. In both locations, the imaging data is stored in a series of tar files that can be directly downloaded through a HTTP client (e.g., a web browser, Curl or wget). The

data is additionally available on S3 as individual NifTI files for each scan, which can be downloaded using a HTTP client or S3 client software such as Cyberduck [42].

All imaging data are released in the NIfTI file format; they are organized and named according to the brain imaging data structure (BIDS) format [43].

Phenotypic Data

Partial phenotypic data will be publicly available without any requirements for a data usage agreement. This includes age, sex, handedness, the internal state questionnaire, and the New York Cognition Questionnaire [43]. These data are located in a comma separated value (.csv) file accessible via the HBN-SSI website and are included with the BIDS-organized imaging data as tab separate values (TSV) files. The remainder of the phenotypic data (see Table 3), including the PANAS [44] and results from the ADHD Quotient system [45], will be made available to investigators following completion of the HBN Data Usage Agreement (DUA). The HBN DUA is modeled after that of the NKI-Rockland Sample and is intended to prevent against data re-identification; it does not place any constraints on the range of analyses that can be carried out using the shared data, or place requirements for co-authorship. Following submission and execution of the data usage agreement, users can access the phenotypic data through the COINS Data Exchange (an enhanced graphical query tool, which enables users to target and download files in accord with specific search criteria) [46].

[Table 3 – Questionnaires and physical measures collected.]

TECHNICAL VALIDATION

Quality Assessment

Consistent with the established FCP/INDI policy, all completed datasets contributed to HBN-SSI are made available to users regardless of data quality. Justifications for this decision include the lack of consensus within the imaging community on what constitutes good or poor quality data, and the utility of 'lower quality' datasets for facilitating the development of artifact correction techniques. For HBN-SSI, the inclusion of datasets with significant artifacts related to factors such as motion are particularly valuable, as it facilitates the evaluation of the impact of such real-world confounds on reliability and reproducibility.

To help users assess data quality, we calculated a variety of quantitative quality metrics from the data using the Preprocessed Connectome Project Quality Assurance Protocol (QAP) [47]. The QAP includes a broad range of quantitative metrics that have been proposed in the imaging literature for assessing data quality [48].

For the structural data, spatial measures include: Signal-to-Noise Ratio (SNR) [49], Contrast-to-Noise Ratio (CNR) [49], Foreground-to-Background Energy Ratio (FBER), Percent artifact voxels (QI1) [50], Spatial smoothness (FWHM) [51], Entropy focus criterion (EFC) [52]. These are shown for different participants in Figure 2. Spatial measures of fMRI data include (Figure 3): EFC, FBER, FWHM, and well as Ghost-to-Signal Ratio (GSR) [53]. Temporal measures of fMRI data include (Figure 4): Mean Frame-wise Displacement (Mean FD) [54], Median Distance Index (Quality) [55], Standardized DVARS (DVARS) [56], Outliers Detection [55], and Global correlation (GCOR) [57]. See Figures 2-4 for a subset of the metrics; the full set of measures are included on the HBN-SSI website in .csv format for download. Review of the QAP profiles led us to exclude 3 participants based on excessively high mean FD from the illustrative analyses presented in the next section. Although not a

focus of the current work, visual inspection of the figures points to the potential value of this dataset for establishing the reliability of QAP measures. The impact of scan condition on each of the functional QAP measures was examined using a one-way ANOVA. No significant differences were found for any of the measures. In addition, the test-retest reliability of each QAP measure, for each condition, was assessed using the intra-class correlation coefficient (ICC). The results are shown in Table 4.

Table 4 – ICC values representing the test-retest reliability of Quality Assurance Protocol (QAP) measures, for each scan condition.

Measure	Rest	Inscapes	Movie	Flanker
EFC	0.90	0.91	0.93	0.92
FBER	0.84	0.84	0.84	0.83
FWHM	0.58	0.60	0.74	0.76
GSR	0.56	0.56	0.61	0.62
SNR	0.92	0.91	0.93	0.92
Outliers	0.08	0.18	0.06	0.50
GCOR	0.11	0.09	0.16	0.04
Quality	0.94	0.94	0.93	0.95
Mean FD	0.30	0.39	0.40	0.68
DVARS	0.42	0.49	0.47	0.49

FMRI Analyses. A broad range of analyses, including but not limited to evaluations of test-retest reliability, can be performed using the present HBN-SSI dataset. Here, we provide a few illustrative analyses to demonstrate the technical validity and utility of these data; they are not intended to be exhaustive.

Data preprocessing.

Prior to image processing, Freesurfer was used to combine the 12 available MPRAGE images into an MRI robust average image for each individual participant. A non-rigid registration between MPRAGE images and a 2mm MNI brain-only template (FSL's MNI152_T1_2mm_brain.nii.gz, [58]) was calculated using ANTs [59]. Further anatomical processing included with skull stripping using AFNI's 3dSkullstrip [60] (to include any voxels in the ventricles incorrectly removed by this utility, the brain mask was augmented using a ventricle mask that was generated by reverse transforming the ventricles included in the MNI atlas into native space for each participant). Next, data was processed using a development version of the open-source, Nipype-based [61]- Configurable Pipeline for the Analysis of Connectomes [1] (C-PAC version 0.4.0 [62]). See here for image preprocessing configuration file [63].

Following resampling of the functional MRI data to RPI orientation, image preprocessing in C-PAC consisted of the following steps: 1) motion correction, 2) boundary-based registration [64], 3) nuisance variable regression (1st and 2nd order polynomial, 24-regressor model of motion [65], mean WM mask signal, mean CSF mask signal). We then extracted representative time series for each ROI in the CC200 atlas [66] (by averaging within-ROI voxel time series). All possible pairwise correlations were calculated amongst ROI time series to generate a ROI-to-ROI connectivity matrix for each scan in each session for each subject. To facilitate ease of presentation and interpretation for our findings, the connections were sorted by intrinsic connectivity network membership, as defined by Yeo et al. [67].

Fingerprinting. Prior work by Finn et al. [22] demonstrated the ability to "fingerprint" individuals based on their functional connectivity matrices. Specifically, they found that the level of correlation between connectivity matrices for data obtained from the same participant on different occasions was markedly higher than that observed for connectivity

matrices obtained from different participants; this was true regardless of whether functional connectivity was based on resting state or task activation data. Consistent with their work, we found a dramatically higher degree of correlation, using Pearson's R, between connectivity matrices obtained from the same individual on differing sessions (Mean: 0.599, Standard Deviation: 0.083, 95% Confidence Interval: 0.598 – 0.600), when compared to differing individuals (Mean: 0.445, Standard Deviation: 0.065, 95% Confidence Interval: 0.444 – 0.445) (Figure 5).. Also consistent with their findings, we found this to be true regardless of the scan condition employed.

Connection-Wise Reliability For the Four States. A key question is how much variation among scan conditions (i.e., between-condition reliability) impacts reliability as opposed to between-session reliability (i.e., test-retest reliability). To address this question, we analyzed the 12 sessions obtained for the 10 participants with minimal head motion using a hierarchical Linear Mixed Model (*note:* three subjects were missing the flanker task from one session each; these were treated as missing values in our analyses). The hierarchical LMM allows for the estimation of reliability by providing estimates of variance between participants, across the four conditions (for the same participant) and between sessions within each condition.

$$iFC_{ijk}(v) = \mu_{000}(v) + \gamma_{jk}(v) + \delta_k(v) + \varepsilon_{ijk}(v) \qquad (1)$$

For a given functional connectivity measurement ν, $iFC_{ijk}(\nu)$ is the modeled intrinsic functional connectivity for the i-th session, for the j-th condition of the k-th participant, taking into account condition and session effects. The equation is composed of an intercept μ_{000}, a random effect between sessions for the j-th condition of k-th participant γ_{jk}, a random effect for the k-th participant δ_k, and an error term ε_{ijk}.γ_{jk}, δ_k, and ε_{ijk} are assumed to be independent, and follow a normal distribution with zero mean. The total variances of iFC

can be decomposed into three parts, 1) variance between participants (σ_3^2= Var[δ]) , 2) variance between conditions for the same participant (σ_2^2 = Var[γ]), and 3) variance of the residual; indicating variance between sessions (σ_0^2= Var[ϵ]). . The reliability of the iFC across conditions can be calculated as intra-class correlation coefficients as follows (Figure 6, left):

$$ICC(between - conditions) = \frac{\sigma_3^2}{\sigma_3^2+\sigma_2^2} \qquad (2)$$

and across sessions as follows Figure 6, right)):

$$ICC(between - sessions, conditions) = \frac{\sigma_3^2+\sigma_2^2}{\sigma_3^2+\sigma_2^2+\sigma_0^2} \qquad (3)$$

Findings revealed impressively high degree of between-condition reliability for most connections (percentiles: 50th: 0.854, 75th: 0.955, 95th: 1), as opposed to between-session (i.e., test-retest) reliability, which was notably lower (percentiles: 50th: 0.270, 75th: 0.355, 95th: 0.507). Of interest, between-condition reliability tended to be lowest in the visual and somatosensory networks – each of which would be expected to vary in a systematic way across conditions due to differences in visual stimulation (movie > inscapes > flanker > rest) and motor demands (flanker > all other conditions).

Regarding test-retest reliability, follow-up analyses also looked at connection-wise ICC for each of the stimulus/task conditions separately using a linear mixed model (as implemented in R) (see Figure 7), finding similar ranges of ICC scores across conditions, though with some notable differences (e.g., higher ICC for visual network in movies and inscapes; higher frontoparietal ICC's in flanker task and rest). Table 5 gives a breakdown of the summary statistics for each scan condition, for within network conditions and between network

connections. Additionally, we used image-wise correlation coefficient (I2C2) [68] to look at functional networks and their interactions from a multivariate perspective. As can be seen in Figure 7, a high degree of correspondence was noted between the strength of the reliability for a given network (i.e., I2C2) and the strengths of the reliabilities for the individual edges in the network (i.e., ICC).

Table 5 – Displayed here are summary statistics of the distribution of ICC values from the test-retest reliability analysis of each scan condition. Shown are the mean, standard deviation (Std. Dev.), and 95% confidence interval (95% C.I.) of ICC values for within network and between network connections.

	Within Network				Between Network			
	Mean	Std. Dev.	95% C.I.		Mean	Std. Dev.	95% C.I.	
Rest	0.349	0.148	0.345	0.352	0.272	0.130	0.268	0.276
Inscapes	0.332	0.152	0.328	0.336	0.218	0.127	0.214	0.222
Movie	0.356	0.151	0.352	0.360	0.261	0.125	0.257	0.265
Flanker	0.366	0.178	0.362	0.371	0.277	0.148	0.272	0.282

Finally, to gain insights into the effects of scan duration on test-retest reliabilities, we repeated ICC and I2C2 analyses using 10, 20 and 30 minutes of scan data across 4 pseudo-sessions (i.e., for 20 minutes, we combined data from 2 sessions; for 30 minutes, we combined data from 3 sessions). Consistent with prior reports, our analyses revealed notable improvement of ICC and I2C2 values with longer scans, particularly when increasing from 20 to 30 minutes (see Figures 8, 9).

Concluding Remarks. These illustrative analyses highlight the value of these data for addressing questions regarding between-condition and between-session reliability. Beyond

quantifying reliabilities for connectomic indices, the data available can also be used by investigators to answer questions regarding minimum data requirements (e.g., number of timepoints) and optimal image processing strategies. Finally, it is worth noting that the availability of naturalistic viewing states (Inscapes, movie clips) in the resource will give resting state fMRI-focused investigators an opportunity to explore the added value of these states for calculating intrinsic functional connectivity and more (e.g., exploration of inter-subject correlation and inter-subject functional connectivity [23], [69]).

SOFTWARE AND AVAILABILITY

The Configurable Pipeline for the Analysis of Connectomes (C-PAC) was employed to carry out the image processing for the analyses included in the text, and can be downloaded from the C-PAC webpage [62]. Additionally, the configuration file containing the settings for C-PAC is available for download [63]. C-PAC is a python based software, which can run on Unix based platforms. Windows is not supported. Note, not all of C-PAC's dependencies are python based. A list of the dependencies can be found on the C-PAC webpage under the installation section [70]. C-PAC operates under a BSD 3-Clause license. Snapshots of the C-PAC code and other supporting metadata are openly available in the GigaScience repository, GigaDB [71].

AVAILABILITY OF SUPPORTING DATA

The HBN-SSI is available online [36]. Further supporting metadata are openly available in the GigaScience repository, GigaDB [71].

LIST OF ABBREVIATIONS

HBN – Healthy Brain Network

SSI – Serial Scanning Initiative

R-fMRI – Resting State Functional Magnetic Resonance Imaging

DKI – Diffusion Kurtosis Imaging

MPRAGE – Magnetization Prepared Rapidly Acquired Gradient Echo

MNI – Montreal Neurological Institute

FSL – FMRIB Software Library

AFNI – Analysis of Functional NeuroImages

ANTs – Advanced Normalization Tools

iFC – Intrinsic Functional Connectivity

ICN – Intrinsic Connectivity Network

CPAC – Configurable Pipeline for Analysis of Connectomes

QAP – Quality Assurance Protocol

SNR - Signal-to-Noise Ratio

CNR - Contrast-to-Noise Ratio

FBER - Foreground-to-Background Energy Ratio

QI1 - Percent artifact voxels

FWHM – Full Width Half Maximum

EFC - Entropy focus criterion

GSR - Ghost-to-Signal Ratio

Mean FD - Mean Frame-wise Displacement

GCOR - Global correlation

ICC – Intra-class Correlation Coefficient

I2C2 – Image Intra-class Correlation Coefficient

ETHICS APPROVAL AND CONSENT TO PARTICIPATE

All experimental procedures were performed with approval of the Chesapeake Institutional Review Board and only after informed consent were obtained.

CONSENT FOR PUBLICATION

All participants consented to have their data shared.

COMPETING INTERESTS

The authors declare that they have no competing interests.

FUNDING

This work was supported by The Healthy Brain Network (http://www.healthybrainnetwork.org) and its supporting initiatives are supported by philanthropic contributions from the following individuals, foundations and organizations: Lee Alexander, Robert Allard, Lisa Bilotti Foundation, Inc., Margaret Billoti, Christopher Boles, Brooklyn Nets, Agapi and Bruce Burkhard, Randolph Cowen and Phyllis Green, Elizabeth and David DePaolo, Charlotte Ford, Valesca Guerrand-Hermes, Sarah and Geoffrey Gund, George Hall, Joseph Healey and Elaine Thomas, Hearst Foundations, Eve and Ross Joffe, Anton and Robin Katz, Rachael and Marshall Levine, Ke Li, Jessica Lupovici, Javier Macaya, Christine and Richard Mack, Susan Miller and Byron Grote, John and Amy Phelan, Linnea and George Roberts, Jim and Linda Robinson Foundation, Inc, Caren and Barry Roseman, Zibby Schwarzman, David Shapiro and Abby Pogrebin, Stavros Niarchos Foundation, Nicholas Van Dusen, David Wolkoff and Stephanie Winston Wolkoff and the Donors to the Brant Art Auction of 2012.

AUTHOR CONTRIBUTIONS

Conception and Experimental Design:

JE, LP, MPM, RCC, SC, SG, TV

Implementation and Logistics:

DOC, NVP, RCC, SC, TV

Data Collection:

AB, MK, NGV, YO

Data Informatics:

DOC, JP, RCC

Data Analysis:

DOC, LA, MPM, TX

Drafting of the Manuscript:

DOC, MPM, RCC, TX

Critical Review and Editing of the Manuscript:

All Authors contributed equally to the critical review and editing of the manuscript.

ACKNOWLEDGEMENTS

AUTHOR DETAILS

1. Child Mind Institute Healthy Brain Network, New York, New York
2. Center for Biomedical Imaging and Neuromodulation, Nathan S. Kline Institute for Psychiatric Research, Orangeburg, New York

3. Yale University, New Haven, Connecticut

4. City College of New York, New York, New York

5. The Graduate Center of the City University of New York, New York, New York

6. Massachusetts Institute of Technology, Cambridge, Massachusetts

Bibliography

[1] R. C. Craddock, S. Jbabdi, C.-G. Yan, J. T. Vogelstein, F. X. Castellanos, A. Di Martino, C. Kelly, K. Heberlein, S. Colcombe, and M. P. Milham, "Imaging human connectomes at the macroscale," *Nat Meth*, vol. 10, no. 6, pp. 524–539, Jun. 2013.

[2] C. Kelly, B. B. Biswal, R. C. Craddock, F. X. Castellanos, and M. P. Milham, "Characterizing variation in the functional connectome: promise and pitfalls," *Trends Cogn. Sci.*, vol. 16, no. 3, pp. 181–188, Mar. 2012.

[3] O. Sporns, "The human connectome: a complex network," *Ann. N. Y. Acad. Sci.*, vol. 1224, no. 1, pp. 109–125, 2011.

[4] E. T. Bullmore and D. S. Bassett, "Brain Graphs: Graphical Models of the Human Brain Connectome," *Annu. Rev. Clin. Psychol.*, vol. 7, no. 1, pp. 113–140, Mar. 2011.

[5] S. M. Smith, D. Vidaurre, C. F. Beckmann, M. F. Glasser, M. Jenkinson, K. L. Miller, T. E. Nichols, E. C. Robinson, G. Salimi-Khorshidi, M. W. Woolrich, D. M. Barch, K. Uğurbil, and D. C. Van Essen, "Functional connectomics from resting-state fMRI," *Trends Cogn. Sci.*, vol. 17, no. 12, pp. 666–682, Dec. 2013.

[6] R. L. Buckner, F. M. Krienen, and B. T. T. Yeo, "Opportunities and limitations of intrinsic functional connectivity MRI," *Nat Neurosci*, vol. 16, no. 7, pp. 832–837, Jul. 2013.

[7] K. R. A. Van Dijk, T. Hedden, A. Venkataraman, K. C. Evans, S. W. Lazar, and R. L. Buckner, "Intrinsic Functional Connectivity As a Tool For Human Connectomics: Theory, Properties, and Optimization," *J. Neurophysiol.*, vol. 103, no. 1, p. 297 LP-

321, Jan. 2010.

[8] Z. Shehzad, A. M. C. Kelly, P. T. Reiss, D. G. Gee, K. Gotimer, L. Q. Uddin, S. H. Lee, D. S. Margulies, A. K. Roy, B. B. Biswal, E. Petkova, F. X. Castellanos, and M. P. Milham, “The Resting Brain: Unconstrained yet Reliable,” *Cereb. Cortex* , vol. 19, no. 10, pp. 2209–2229, Oct. 2009.

[9] X.-N. Zuo and X.-X. Xing, “Test-retest reliabilities of resting-state FMRI measurements in human brain functional connectomics: A systems neuroscience perspective,” *Neurosci. Biobehav. Rev.*, vol. 45, pp. 100–118, 2014.

[10] X.-N. Zuo, J. S. Anderson, P. Bellec, R. M. Birn, B. B. Biswal, J. Blautzik, J. C. S. Breitner, R. L. Buckner, V. D. Calhoun, F. X. Castellanos, A. Chen, B. Chen, J. Chen, X. Chen, S. J. Colcombe, W. Courtney, R. C. Craddock, A. Di Martino, H.-M. Dong, X. Fu, Q. Gong, K. J. Gorgolewski, Y. Han, Y. He, Y. He, E. Ho, A. Holmes, X.-H. Hou, J. Huckins, T. Jiang, Y. Jiang, W. Kelley, C. Kelly, M. King, S. M. LaConte, J. E. Lainhart, X. Lei, H.-J. Li, K. Li, K. Li, Q. Lin, D. Liu, J. Liu, X. Liu, Y. Liu, G. Lu, J. Lu, B. Luna, J. Luo, D. Lurie, Y. Mao, D. S. Margulies, A. R. Mayer, T. Meindl, M. E. Meyerand, W. Nan, J. A. Nielsen, D. O’Connor, D. Paulsen, V. Prabhakaran, Z. Qi, J. Qiu, C. Shao, Z. Shehzad, W. Tang, A. Villringer, H. Wang, K. Wang, D. Wei, G.-X. Wei, X.-C. Weng, X. Wu, T. Xu, N. Yang, Z. Yang, Y.-F. Zang, L. Zhang, Q. Zhang, Z. Zhang, Z. Zhang, K. Zhao, Z. Zhen, Y. Zhou, X.-T. Zhu, and M. P. Milham, “An open science resource for establishing reliability and reproducibility in functional connectomics,” *Sci. Data*, vol. 1, p. 140049, Dec. 2014.

[11] S. Mueller, D. Wang, M. D. Fox, R. Pan, J. Lu, K. Li, W. Sun, R. L. Buckner, and H. Liu, “Reliability correction for functional connectivity: Theory and implementation.,” *Hum. Brain Mapp.*, vol. 36, no. 11, pp. 4664–80, Nov. 2015.

[12] D. G. Tomasi, E. Shokri-Kojori, and N. D. Volkow, “Temporal Evolution of Brain Functional Connectivity Metrics: Could 7 Min of Rest be Enough?,” *Cereb. Cortex* ,

Aug. 2016.

[13] F. X. Castellanos, A. Di Martino, R. C. Craddock, A. D. Mehta, and M. P. Milham, “Clinical applications of the functional connectome,” *Neuroimage*, vol. 80, pp. 527–540, 2013.

[14] A. Zalesky, A. Fornito, and E. T. Bullmore, “Network-based statistic: Identifying differences in brain networks,” *Neuroimage*, vol. 53, no. 4, pp. 1197–1207, 2010.

[15] T. Vanderwal, C. Kelly, J. Eilbott, L. C. Mayes, and F. X. Castellanos, “Inscapes: A movie paradigm to improve compliance in functional magnetic resonance imaging,” *Neuroimage*, vol. 122, pp. 222–232, 2015.

[16] E. Tagliazucchi and H. Laufs, “Decoding Wakefulness Levels from Typical fMRI Resting-State Data Reveals Reliable Drifts between Wakefulness and Sleep,” *Neuron*, vol. 82, no. 3, pp. 695–708, 2014.

[17] J. D. Power, K. A. Barnes, A. Z. Snyder, B. L. Schlaggar, and S. E. Petersen, “Spurious but systematic correlations in functional connectivity MRI networks arise from subject motion,” *Neuroimage*, vol. 59, no. 3, pp. 2142–2154, 2012.

[18] T. D. Satterthwaite, M. A. Elliott, R. T. Gerraty, K. Ruparel, J. Loughead, M. E. Calkins, S. B. Eickhoff, H. Hakonarson, R. C. Gur, R. E. Gur, and D. H. Wolf, “An improved framework for confound regression and filtering for control of motion artifact in the preprocessing of resting-state functional connectivity data,” *Neuroimage*, vol. 64, pp. 240–256, 2013.

[19] V. Betti, S. Della Penna, F. de Pasquale, D. Mantini, L. Marzetti, G. L. Romani, and M. Corbetta, “Natural scenes viewing alters the dynamics of functional connectivity in the human brain,” *Neuron*, vol. 79, no. 4, pp. 782–797, 2013.

[20] A. Bartels and S. Zeki, “Functional brain mapping during free viewing of natural scenes.,” *Hum. Brain Mapp.*, vol. 21, no. 2, pp. 75–85, Feb. 2004.

[21] M. Mennes, C. Kelly, X.-N. Zuo, A. Di Martino, B. B. Biswal, F. X. Castellanos, and M.

P. Milham, “Inter-individual differences in resting-state functional connectivity predict task-induced BOLD activity,” *Neuroimage*, vol. 50, no. 4, pp. 1690–1701, 2010.

[22] E. S. Finn, X. Shen, D. Scheinost, M. D. Rosenberg, J. Huang, M. M. Chun, X. Papademetris, and R. T. Constable, “Functional connectome fingerprinting: identifying individuals using patterns of brain connectivity,” *Nat. Neurosci.*, 2015.

[23] E. Simony, C. J. Honey, J. Chen, O. Lositsky, Y. Yeshurun, A. Wiesel, and U. Hasson, “Dynamic reconfiguration of the default mode network during narrative comprehension,” *Nat. Commun.*, vol. 7, 2016.

[24] V. D. Calhoun, K. A. Kiehl, and G. D. Pearlson, “Modulation of temporally coherent brain networks estimated using ICA at rest and during cognitive tasks,” *Hum. Brain Mapp.*, vol. 29, no. 7, pp. 828–838, 2008.

[25] A. M. C. Kelly, L. Q. Uddin, B. B. Biswal, F. X. Castellanos, and M. P. Milham, “Competition between functional brain networks mediates behavioral variability,” *Neuroimage*, vol. 39, no. 1, pp. 527–537, Jan. 2008.

[26] D. A. Fair, B. L. Schlaggar, A. L. Cohen, F. M. Miezin, N. U. F. Dosenbach, K. K. Wenger, M. D. Fox, A. Z. Snyder, M. E. Raichle, and S. E. Petersen, “A method for using blocked and event-related fMRI data to study ‘resting state’ functional connectivity,” *Neuroimage*, vol. 35, no. 1, pp. 396–405, 2007.

[27] R. Patriat, E. K. Molloy, T. B. Meier, G. R. Kirk, V. A. Nair, M. E. Meyerand, V. Prabhakaran, and R. M. Birn, “The effect of resting condition on resting-state fMRI reliability and consistency: A comparison between resting with eyes open, closed, and fixated,” *Neuroimage*, vol. 78, pp. 463–473, 2013.

[28] C. W. Eriksen, “The flankers task and response competition: A useful tool for investigating a variety of cognitive problems,” *Vis. cogn.*, vol. 2, no. 2–3, pp. 101–118, 1995.

[29] J. S. Guntupalli, M. Hanke, Y. O. Halchenko, A. C. Connolly, P. J. Ramadge, and J. V

Haxby, “A Model of Representational Spaces in Human Cortex,” *Cereb. Cortex*, 2016.

[30] J. Wang, L. He, H. Zheng, and Z.-L. Lu, “Optimizing the Magnetization-Prepared Rapid Gradient-Echo (MP-RAGE) Sequence,” *PLoS One*, vol. 9, no. 5, p. e96899, May 2014.

[31] H. Lu, J. H. Jensen, A. Ramani, and J. A. Helpern, “Three-dimensional characterization of non-gaussian water diffusion in humans using diffusion kurtosis imaging,” *NMR Biomed.*, vol. 19, no. 2, pp. 236–247, 2006.

[32] C. B. Shaw, J. H. Jensen, R. L. Deardorff, M. V. Spampinato, and J. A. Helpern, “Comparison of Diffusion Metrics Obtained at 1.5 T and 3T in Human Brain With Diffusional Kurtosis Imaging,” *J. Magn. Reson. Imaging*, 2016.

[33] S. C. L. Deoni, T. M. Peters, and B. K. Rutt, “High-resolution T1 and T2 mapping of the brain in a clinically acceptable time with DESPOT1 and DESPOT2,” *Magn. Reson. Med.*, vol. 53, no. 1, pp. 237–241, 2005.

[34] R. I. Grossman, J. M. Gomori, K. N. Ramer, F. J. Lexa, and M. D. Schnall, “Magnetization transfer: theory and clinical applications in neuroradiology.,” *RadioGraphics*, vol. 14, no. 2, pp. 279–290, Mar. 1994.

[35] “Chesapeake IRB.” [Online]. Available: https://www.chesapeakeirb.com. [Accessed: 10-Apr-2016].

[36] “Healthy Brain Network Serial Scanning Intitiative (HBN-SSI).” [Online]. Available: http://fcon_1000.projects.nitrc.org/indi/hbn_ssi. [Accessed: 10-Apr-2016].

[37] S. Moeller, E. Yacoub, C. A. Olman, E. Auerbach, J. Strupp, N. Harel, and K. Ugurbil, “Multiband multislice GE-EPI at 7 tesla, with 16-fold acceleration using partial parallel imaging with application to high spatial and temporal whole-brain fMRI.,” *Magn. Reson. Med.*, vol. 63, no. 5, pp. 1144–1153, May 2010.

[38] A. J. W. van der Kouwe, T. Benner, D. H. Salat, and B. Fischl, “Brain Morphometry with Multiecho MPRAGE,” *Neuroimage*, vol. 40, no. 2, pp. 559–569, Apr. 2008.

[39] S. C. L. Deoni, B. K. Rutt, T. Arun, C. Pierpaoli, and D. K. Jones, “Gleaning multicomponent T1 and T2 information from steady-state imaging data,” *Magn. Reson. Med.*, vol. 60, no. 6, pp. 1372–1387, 2008.

[40] M. Mennes, B. B. Biswal, F. X. Castellanos, and M. P. Milham, “Making data sharing work: The FCP/INDI experience,” *Neuroimage*, vol. 82, pp. 683–691, Nov. 2013.

[41] A. Bischoff-Grethe, I. B. Ozyurt, E. Busa, B. T. Quinn, C. Fennema-Notestine, C. P. Clark, S. Morris, M. W. Bondi, T. L. Jernigan, A. M. Dale, G. G. Brown, and B. Fischl, “A Technique for the Deidentification of Structural Brain MR Images,” *Hum. Brain Mapp.*, vol. 28, no. 9, pp. 892–903, Sep. 2007.

[42] “CyberDuck.” [Online]. Available: https://cyberduck.io. [Accessed: 10-Apr-2016].

[43] K. J. Gorgolewski, T. Auer, V. D. Calhoun, R. C. Craddock, S. Das, E. P. Duff, G. Flandin, S. S. Ghosh, T. Glatard, Y. O. Halchenko, D. A. Handwerker, M. Hanke, D. Keator, X. Li, Z. Michael, C. Maumet, B. N. Nichols, T. E. Nichols, J. Pellman, J.-B. Poline, A. Rokem, G. Schaefer, V. Sochat, W. Triplett, J. A. Turner, G. Varoquaux, and R. A. Poldrack, “The brain imaging data structure, a format for organizing and describing outputs of neuroimaging experiments,” *Sci. Data*, vol. 3, p. 160044, Jun. 2016.

[44] D. Watson, L. A. Clark, and A. Tellegen, “Development and validation of brief measures of positive and negative affect: The PANAS scales.,” *Journal of Personality and Social Psychology*, vol. 54, no. 6. American Psychological Association, US, pp. 1063–1070, 1988.

[45] C. R. Sumner, “New Tool for Objective Assessments of ADHD: The Quotient™ ADHD System,” *ADHD Rep.*, vol. 18, no. 5, pp. 6–9, Oct. 2010.

[46] A. Scott, W. Courtney, D. Wood, R. la Garza, S. Lane, R. Wang, M. King, J. Roberts, J. Turner, and V. Calhoun, “COINS: An Innovative Informatics and Neuroimaging Tool Suite Built for Large Heterogeneous Datasets,” *Front. Neuroinform.*, vol. 5, p. 33,

2011.

[47] "Quality Assurance Protocol." [Online]. Available: http://preprocessed-connectomes-project.org/quality-assessment-protocol. [Accessed: 10-Apr-2016].

[48] Z. Shehzad, S. Giavasis, Q. Li, Y. Benhajali, C. Yan, Z. Yang, M. Milham, P. Bellec, and C. Craddock, "The Preprocessed Connectomes Project Quality Assessment Protocol-a resource for measuring the quality of MRI data."

[49] V. A. Magnotta and L. Friedman, "Measurement of Signal-to-Noise and Contrast-to-Noise in the fBIRN Multicenter Imaging Study," *J. Digit. Imaging*, vol. 19, no. 2, pp. 140–147, 2006.

[50] B. Mortamet, M. A. Bernstein, C. R. Jack, J. L. Gunter, C. Ward, P. J. Britson, R. Meuli, J.-P. Thiran, and G. Krueger, "Automatic quality assessment in structural brain magnetic resonance imaging.," *Magn. Reson. Med.*, vol. 62, no. 2, pp. 365–72, Aug. 2009.

[51] L. Friedman, G. H. Glover, D. Krenz, and V. Magnotta, "Reducing inter-scanner variability of activation in a multicenter fMRI study: Role of smoothness equalization," *Neuroimage*, vol. 32, no. 4, pp. 1656–1668, 2006.

[52] D. Atkinson, D. L. G. Hill, P. N. R. Stoyle, P. E. Summers, and S. F. Keevil, "Automatic correction of motion artifacts in magnetic resonance images using an entropy focus criterion," *IEEE Trans. Med. Imaging*, vol. 16, no. 6, pp. 903–910, Dec. 1997.

[53] M. Giannelli, S. Diciotti, C. Tessa, and M. Mascalchi, "Characterization of Nyquist ghost in EPI-fMRI acquisition sequences implemented on two clinical 1.5 T MR scanner systems: effect of readout bandwidth and echo spacing," *J. Appl. Clin. Med. Phys.*, vol. 11, no. 4, 2010.

[54] M. Jenkinson, P. Bannister, M. Brady, and S. Smith, "Improved Optimization for the Robust and Accurate Linear Registration and Motion Correction of Brain Images,"

Neuroimage, vol. 17, no. 2, pp. 825–841, 2002.

[55] R. W. Cox, "AFNI: Software for Analysis and Visualization of Functional Magnetic Resonance Neuroimages," *Comput. Biomed. Res.*, vol. 29, no. 3, pp. 162–173, 1996.

[56] T. Nichols, "Notes on creating a standardized version of DVARS," 2013.

[57] Z. S. Saad, R. C. Reynolds, H. J. Jo, S. J. Gotts, G. Chen, A. Martin, and R. W. Cox, "Correcting brain-wide correlation differences in resting-state FMRI," *Brain Connect.*, vol. 3, no. 4, pp. 339–352, 2013.

[58] S. M. Smith, M. Jenkinson, M. W. Woolrich, C. F. Beckmann, T. E. J. Behrens, H. Johansen-Berg, P. R. Bannister, M. De Luca, I. Drobnjak, D. E. Flitney, R. K. Niazy, J. Saunders, J. Vickers, Y. Zhang, N. De Stefano, J. M. Brady, and P. M. Matthews, "Advances in functional and structural MR image analysis and implementation as FSL," *Neuroimage*, vol. 23, pp. S208–S219, 2004.

[59] B. B. Avants, N. Tustison, and G. Song, "Advanced normalization tools (ANTS)," *Insight J*, vol. 2, pp. 1–35, 2009.

[60] M. Jenkinson, M. Pechaud, and S. Smith, "BET2: MR-based estimation of brain, skull and scalp surfaces," in *Eleventh annual meeting of the organization for human brain mapping*, 2005, vol. 17, p. 167.

[61] K. Gorgolewski, C. D. Burns, C. Madison, D. Clark, Y. O. Halchenko, M. L. Waskom, and S. S. Ghosh, "Nipype: a flexible, lightweight and extensible neuroimaging data processing framework in python," *Front. Neuroinform.*, vol. 5, p. 13, 2011.

[62] "Configurable Pipeline for the Analysis of Connectomes." [Online]. Available: http://fcp-indi.github.io. [Accessed: 10-Apr-2016].

[63] "C-PAC Configuration file." [Online]. Available: https://www.nitrc.org/frs/downloadlink.php/9275. [Accessed: 10-Apr-2016].

[64] D. N. Greve and B. Fischl, "Accurate and robust brain image alignment using boundary-based registration," *Neuroimage*, vol. 48, no. 1, pp. 63–72, 2009.

[65] K. J. Friston, S. Williams, R. Howard, R. S. J. Frackowiak, and R. Turner, “Movement-Related effects in fMRI time-series,” *Magn. Reson. Med.*, vol. 35, no. 3, pp. 346–355, Mar. 1996.

[66] R. C. Craddock, G. A. James, P. E. Holtzheimer, X. P. Hu, and H. S. Mayberg, “A whole brain fMRI atlas generated via spatially constrained spectral clustering,” *Hum. Brain Mapp.*, vol. 33, no. 8, p. 10.1002/hbm.21333, Aug. 2012.

[67] B. T. Thomas Yeo, F. M. Krienen, J. Sepulcre, M. R. Sabuncu, D. Lashkari, M. Hollinshead, J. L. Roffman, J. W. Smoller, L. Zöllei, J. R. Polimeni, B. Fischl, H. Liu, and R. L. Buckner, “The organization of the human cerebral cortex estimated by intrinsic functional connectivity,” *J. Neurophysiol.*, vol. 106, no. 3, p. 1125 LP-1165, Sep. 2011.

[68] H. Shou, A. Eloyan, S. Lee, V. Zipunnikov, A. N. Crainiceanu, M. B. Nebel, B. Caffo, M. A. Lindquist, and C. M. Crainiceanu, “Quantifying the reliability of image replication studies: The image intraclass correlation coefficient (I2C2),” *Cogn. Affect. {&} Behav. Neurosci.*, vol. 13, no. 4, pp. 714–724, 2013.

[69] C. Tailby, R. A. J. Masterton, J. Y. Huang, G. D. Jackson, and D. F. Abbott, “Resting state functional connectivity changes induced by prior brain state are not network specific,” *Neuroimage*, vol. 106, pp. 428–440, 2015.

[70] “C-PAC Dependencies.” [Online]. Available: http://fcp-indi.github.io/docs/user/install.html#instructions-for-installing-particular-dependencies.

[71] D. O’Connor, N. Vega Potler, M. Kovacs, T. Xu, L. Ai, J. Pellman, T. Vanderwal, L. Parra, S. Cohen, S. Ghosh, J. Escalera, N. Grant-Villegas, Y. Osman, A. Bui, R. C. Craddock, and M. P. Milham, “Supporting data for ‘The Healthy Brain Network Serial Scanning Initiative,’” GigaScience Database. 2016. http://dx.doi.org/10.5524/100259.

Figure Legend

Figure 1 – Shown here are sample stimuli from each of the four scan conditions included in the present work. These included: 1) Resting State, (far left), 2) Inscapes (middle left), 3) Movie Clips (e.g., the Matrix; middle right), and 4) Flanker Task (with no-go trials).

Figure 2 - Subset of Quality Assessment Protocol (QAP) spatial anatomical measures for each participant (horizontal axis). Depicted are the following measures: Contrast-to-Noise Ratio (CNR), Signal-to-Noise Ratio (SNR), Entropy Focus Criterion (EFC). Foreground-to-Background Energy Ratio (FBER), Spatial smoothness (FWHM), Percent artifact voxels (QI1). Each point indicates the measure calculated for an individual scan; for each participant, the data t across scan conditions and sessions are depicted using a single color.

Figure 3 - Subset of Quality Assessment Protocol (QAP) spatial functional measures for each participant (horizontal axis). Depicted are the following measures: Ghost to Signal Ratio (GSR), Signal-to-Noise Ratio (SNR), Entropy Focus Criterion (EFC). Foreground-to-Background Energy Ratio (FBER), spatial smoothness (FWHM). Each point indicates the measure calculated for an individual scan; for each participant, the data across scan conditions and sessions are depicted using a single color.

Figure 4 - Subset of Quality Assessment Protocol (QAP) temporal functional measures for each participant (horizontal axis). Depicted are the following measures: Outliers detection (Outliers), Global correlation (GCOR), Quality, Mean Frame-wise Displacement, and Standardized DVARS (DVARS). Each point indicates the measure calculated for an individual scan; for each participant, the data across scan conditions and sessions are depicted using a single color.

Figure 5 - Similarity of full-brain connectivity matrices across participants (green), sessions (blue) and scan conditions (yellow), as measured using Pearson correlation coefficients (red). Also depicted in the bottom right are the distributions of correlation coefficients when

comparing scans from the same subject (Within Subject), and scans from different subjects (Between Subject). The distribution of correlation values is also shown (bottom right). On the right column are the values for scans from the same subject, and on the left are scans from different subjects. The median, first, and third quartiles are also depicted with horizontal lines.

Figure 6 - Intraclass correlation coefficients (ICC) quantifying between-condition reliabilities (left) and between-session reliabilities at the connection-level. ICC values were obtained using a hierarchical linear mixed model. These connection-level values are grouped on the vertical and horizontal axes based membership of Intrinsic Connectivity Networks (ICN). No overlap indicates that the voxel did not spatially overlap with any ICN.

Figure 7- Connection-wise ICC values across all subjects, sessions, and scan conditions (top), as well as network-wise calculations of test-retest reliability carried out using the imagewise intraclass correlation coefficient (I2C2), again across all subjects, sessions and scan conditions (bottom).

Figure 8 – Impact of scan duration on test-retest reliability at the connection level. We randomly sampled sessions, and concatenated the time series temporally to create pseudosessions of 10, 20 and 30 minutes of data. For each of the pseudosession durations, we depict intraclass correlation coefficients (ICC) obtained for each scan condition. *Note:* across durations, the number of pseduosessions was held constant at four.

Figure 9 – Impact of scan duration on test-retest reliability at the network level. We randomly sampled sessions to create pseudosessions of 10, 20 and 30 minutes of data. For each of the pseudosession durations, we depict imagewise intraclass correlation coefficients (I2C2) obtained for each scan condition. *Note:* across durations, the number of pseduosessions was held constant at four.

Tables

Table 3 – Questionnaires and physical measures collected.

Questionnaires	
Internal State Questionnaire (pre-scan, post-scan)	3-item self-report questionnaire assessing hunger and thirst. Participants respond on a visual analogue scale ranging from "I am not hungry/thirsty/full at all" to "I have never been more hungry/thirsty/full". Responses are rated from 0-100. Participants complete this questionnaire before and after each scan.
New York Cognition Questionnaire (NYC-Q) (post-scan)	31-item self-report questionnaire that asks participants about the different thoughts and feelings that they may have had while in the MRI scan. Participants are asked to indicate the extent to which their thinking or experience corresponded to each item on a 9-point scale.
PANAS (post-scan)	The PANAS-S is a self-administered, 20-item Likert scale assessment that measures degree of positive or negative affect. Users are asked to rate 10 adjectives that measure positive feelings such as joy or pleasure, and 10 adjectives that measure negative feelings, such as anxiety or sadness, on a scale of how closely the adjective describes them in the present moment or over the past week. Items are rated on a five-point scale.
Physical Measures	
Vitals	Participant vitals (blood pressure, heart rate, blood glucose level, first day of last menstrual cycle) were collected prior to each scan using standard measurement devices in a laboratory environment.
Voice data samples	Audio samples of participant speech were recorded prior to scanning. Each sample consisted of 10 sentences with 5 different implicit emotions (neutral, happy, sad, angry, fearful), 10 non-words, and 2 minutes of free speech. For each sample different sentences were drawn from the same set of emotions; the non-words also differed in each sample but had similar characteristics (ie number of syllables, chunks). Stimuli were presented on a laptop computer screen. Completion of the sample took up to 15 minutes.
Quotient ADHD System	Quotient is a computer based task designed to assess three core symptoms of ADHD: hyperactivity, attention and impulsivity. Participants respond to stimuli presented with random timing and random placement on a screen. Completion of the task takes up to 30 minutes.

GeneActiv Actimetry Device	Between scanning sessions, participants wore a non-invasive actimetry sensor that recorded heart rate and indices of physical activity and sleep. The device was placed on participants' non-dominant wrist and data was collected at each scanning session.

 Click here to download Table Table2.xlsx

	Structural
Image	Whole Brain T1
Manufacturer	Siemens
Model	Avanto
Head Coil	32 Channel
Field Strength	1.5T
Sequence	3D Despot 1
Flip Angle(s) [Deg]	2.66;3.55;4.44;5.33;6.22;8.0;11.55;16.0
Phase Cycling [Deg]	NA
Inversion Time [ms]	NA
Echo Time [ms]	2.4
Repitition Time [ms]	5.2
Bandwidth per Voxel (Readout) [Hz/Px]	350
Parallel Acquisition	None
Partial Fourier	P6/8 S7/8
Slice Orientation	S
Slice Phase Encoding Direction	AP
Slice Acquisition Order	SA
Slice Gap [%]	20
Field of View [mm]	220x220
Reconstructed Image Matrix	128x128x96
Reconstructed Resolution [mm]	1.72x1.72x1.8
Number of Measurements	8
Acquisition Time [min:sec]	5:00
Fat Supression	None
Number of Directions	NA
Number of B Zeros	NA
B Value (s) [s/mm^2]	NA
Averages	NA

Legend: AP: Anterior Posterior, PA: Posterior Anterior, RL: Right Left, IA: Interleave

Inversion Recovery	Whole Brain T2	Magnetization Transfer
IR-SPGR / 3D Despot 1	3D Despot 2	3D FLASH
5	10.0;13.33;16.66;19.99;23.33;30.0;43.33;60.0	15
NA	0;180	NA
400	NA	NA
2.4	2.7	11
5.3	5.4	30
350	350	350
None	None	GP2
S6/8	P6/8 S7/8	P6/8 S6/8
S	S	S
AP	AP	AP
SA	SA	IA
20	20	20
220x220	220x220	256x256
128x128x48	128x128x96	256x256x176
1.72x1.72x3.6	1.72x1.72x1.8	1.0x1.0
1	16	1
0:53	8:38	6:41
None	None	None
NA	NA	NA
NA	NA	NA
NA	NA	NA
NA	NA	NA

d Ascending, SA: Sequential Ascending, S: Saggital, T: Transverse

ME-MPRAGE RMS	T2 FLAIR	DWI	DKI
ME-MPRAGE/ 3D TFL	FLAIR	EPI	EPI
7	150	90	90
NA	NA	NA	NA
1000	2500	NA	NA
1.64	89	76.2	93.8
2730	9000	3110	4500
651	190	1628	1628
GP2	GP2	MB3	None
None	None	P6/8	P6/8
S	T	T	T
AP	RL	AP/PA	AP
IA	IA	IA	IA
50	30	0	0
256x256	201x230	192x192	192x192
256x256x176	448x512x25	96x96x72	96x96x72
1.0x1.0x1.0	0.45x0.45x6.5	2.0x2.0x2.0	2.0x2.0x2.0
4	1	1	1
6:32	2:44	0:16	9:59
None	On	On	On
NA	NA	64	64
NA	NA	1	1
NA	NA	0	0;1000;2000
1	NA	NA	NA

Functional
Rest/Movie/Inscapes/Flanker
EPI
55
NA
NA
40
1450
2374
MB3
None
T
AP
IA
0
192x192
78x78x54
2.46x2.46x2.5
420
10:18
On
NA
NA
NA
NA

Figure 1 Click here to download Figure figure1.tiff

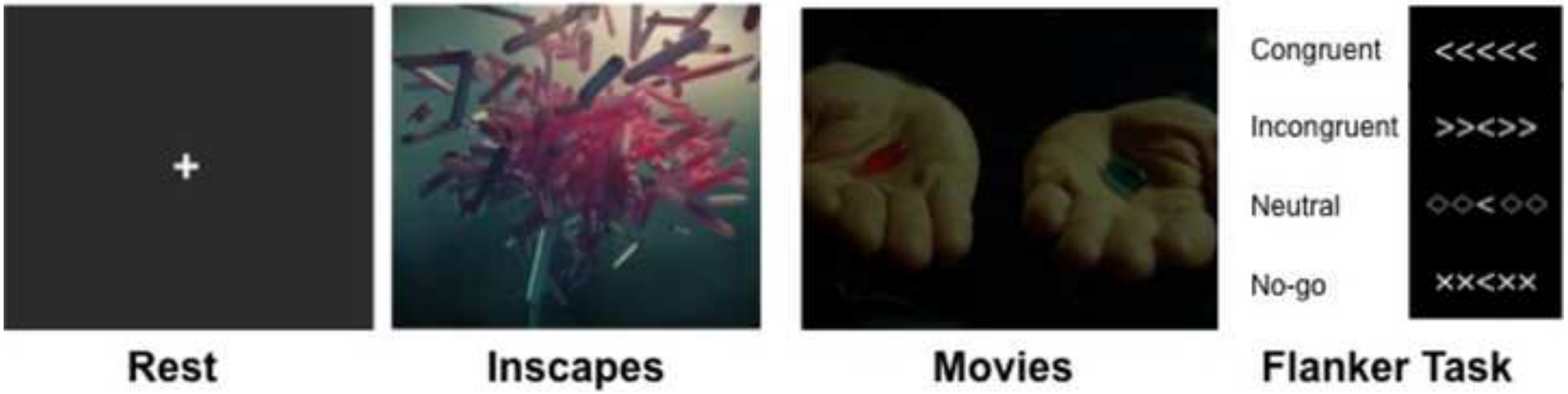

Figure 2 Click here to download Figure figure2.tiff

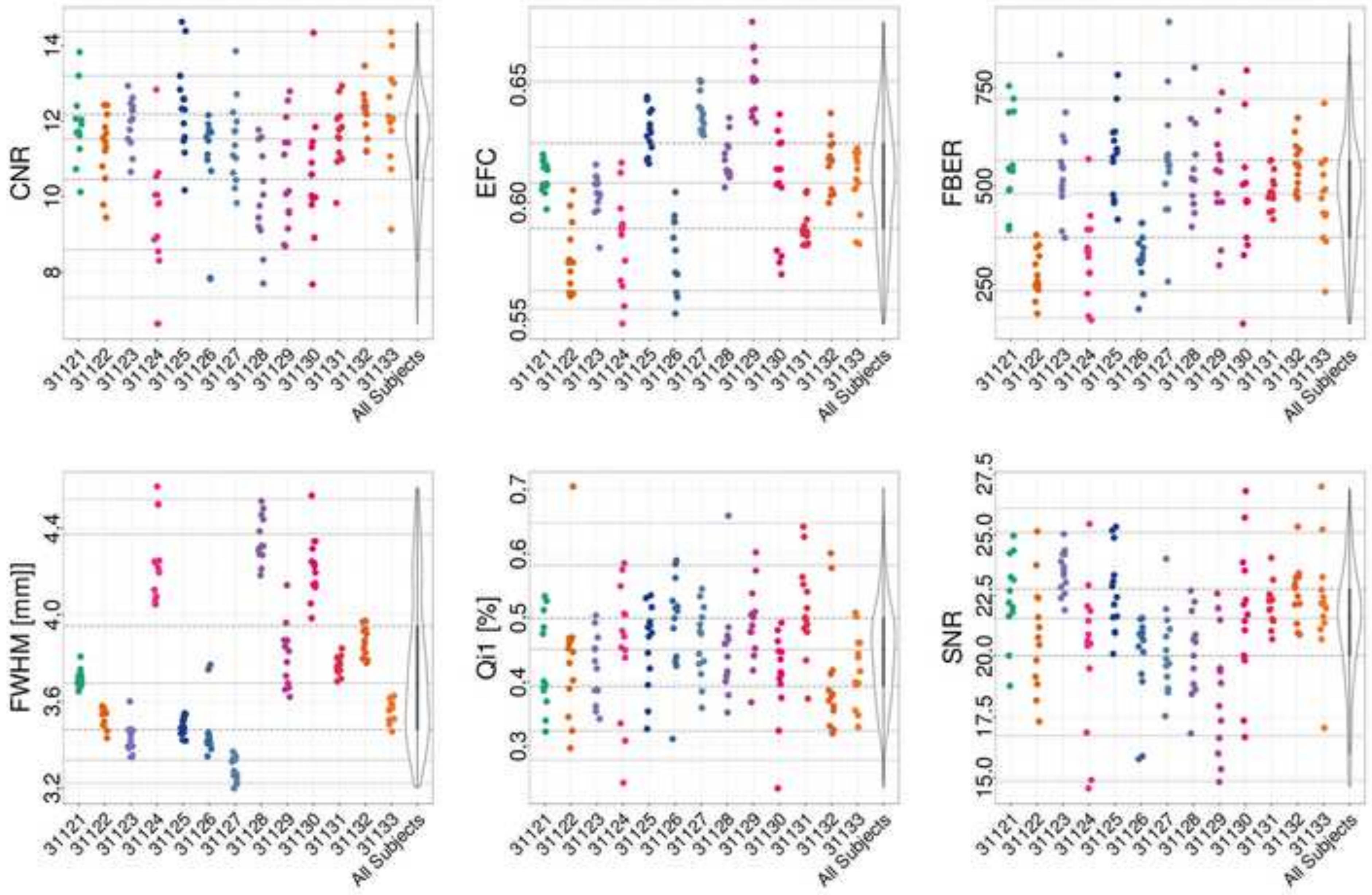

Figure 3

Click here to download Figure figure3.tiff

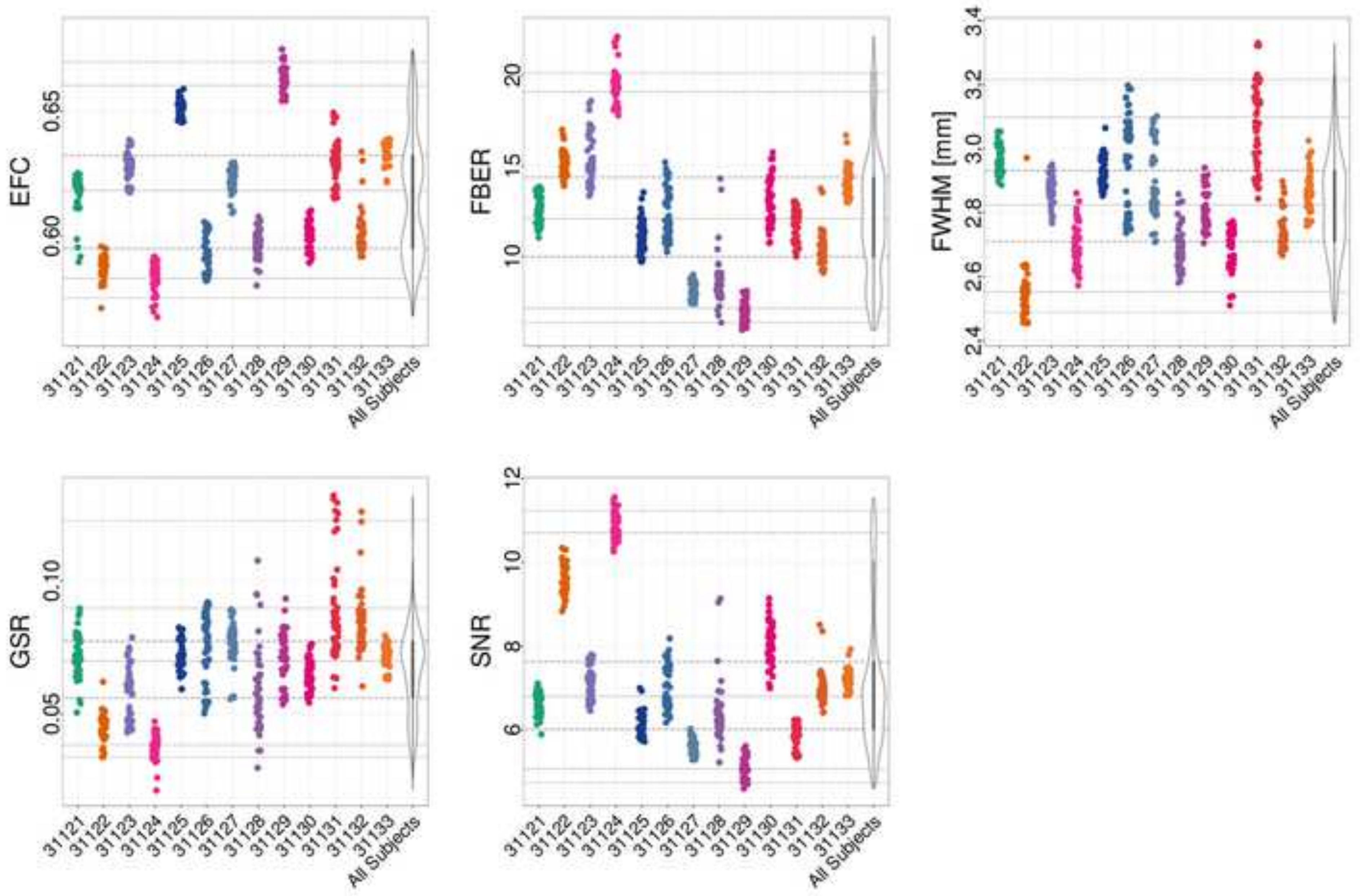

Figure 4

Click here to download Figure figure4.tiff

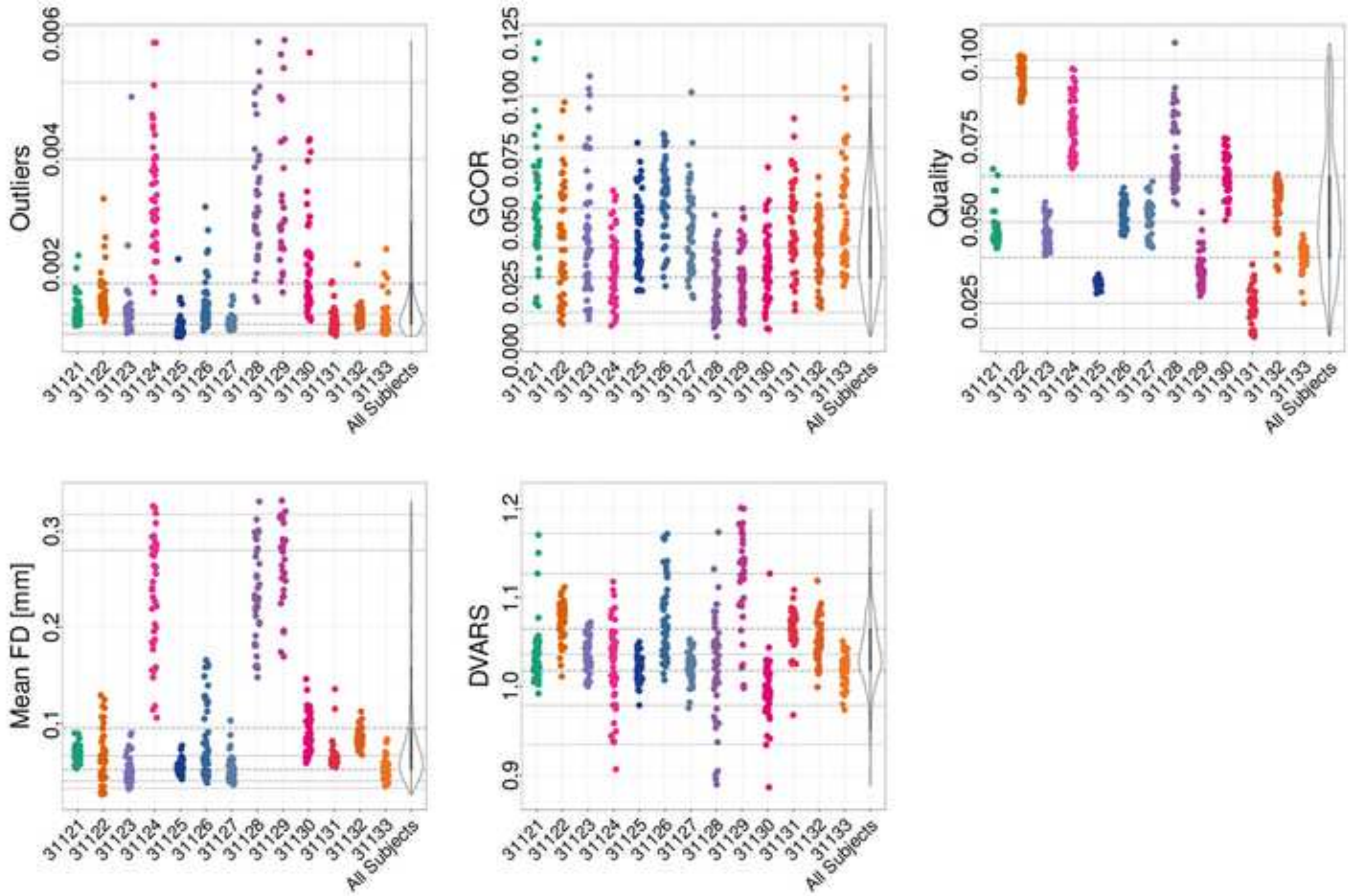

Figure 5

Click here to download Figure figure5.tiff

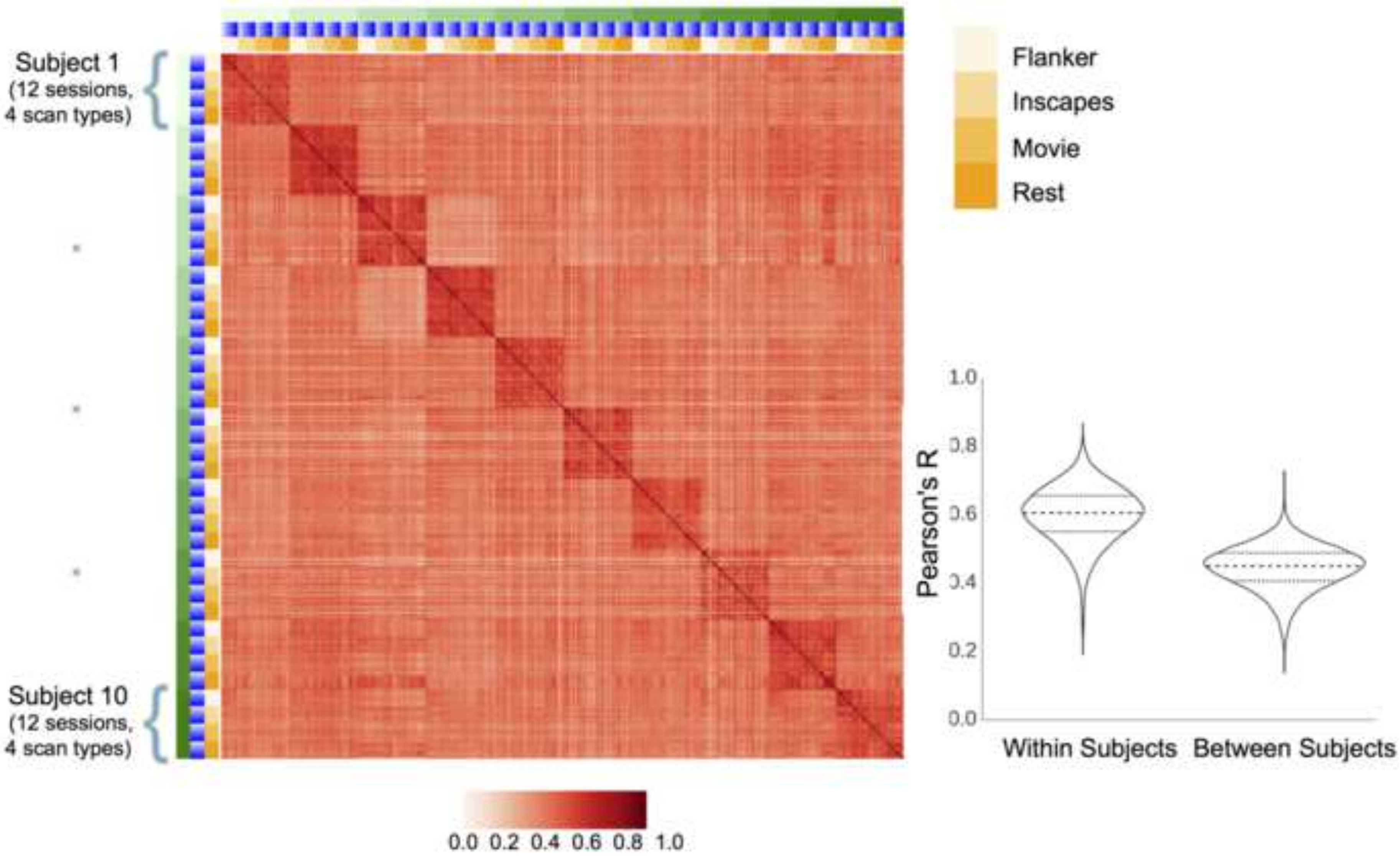

Figure 6

Click here to download Figure figure6.tiff

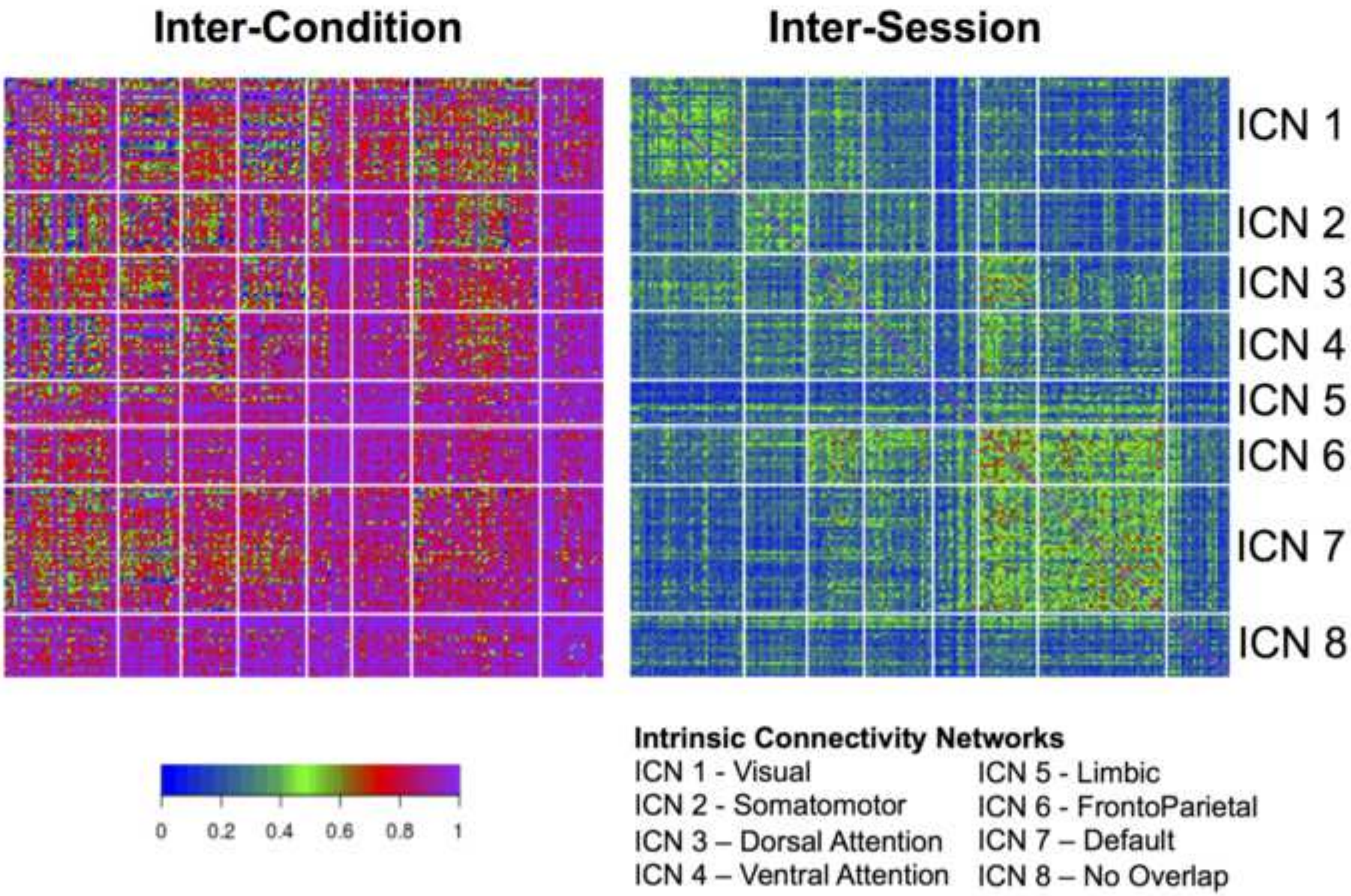

Figure 8

Click here to download Figure figure8.tiff

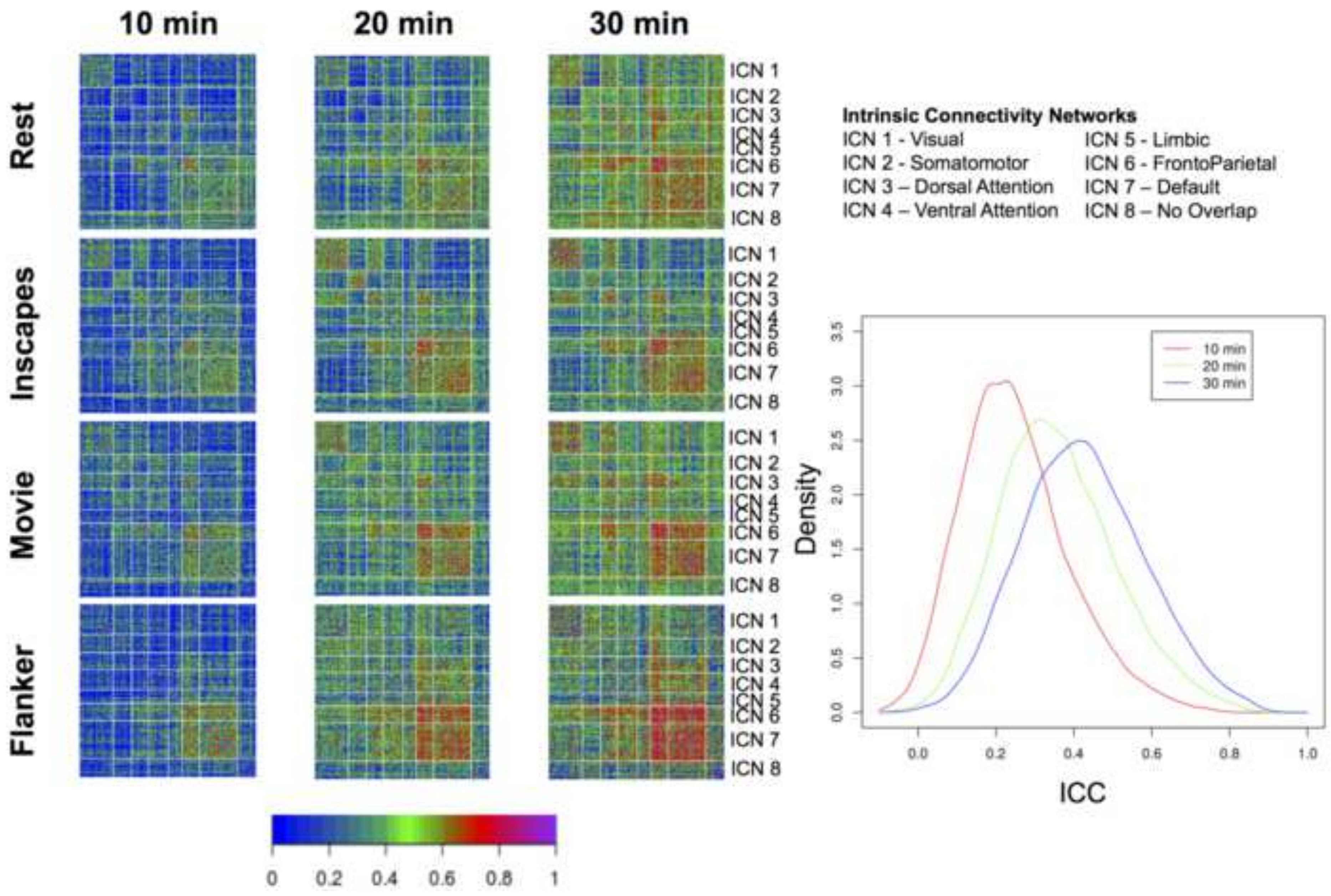

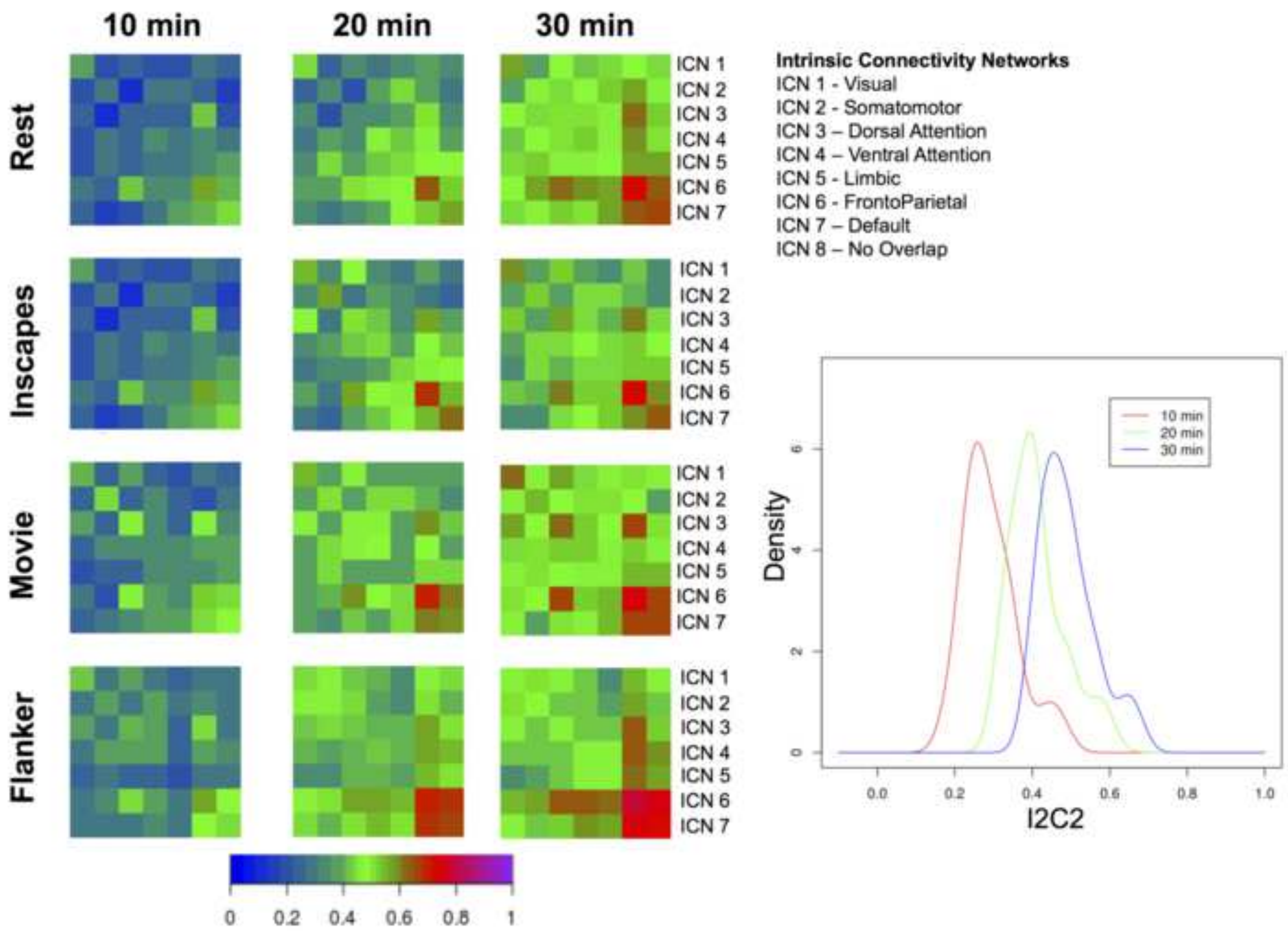
10 min
20 min
30 min
Rest
Inscapes
Movie
Flanker
ICN 1
ICN 2
ICN 3
ICN 4
ICN 5
ICN 6
ICN 7
0
0.2
0.4
0.6
0.8
1
Intrinsic Connectivity Networks
ICN 1 - Visual
ICN 2 - Somatomotor
ICN 3 – Dorsal Attention
ICN 4 – Ventral Attention
ICN 5 - Limbic
ICN 6 - FrontoParietal
ICN 7 – Default
ICN 8 – No Overlap
10 min
20 min
30 min
Density
I2C2
0.0
0.2
0.4
0.6
0.8
1.0
0
2
4
6

 Click here to download Figure figure7.tiff

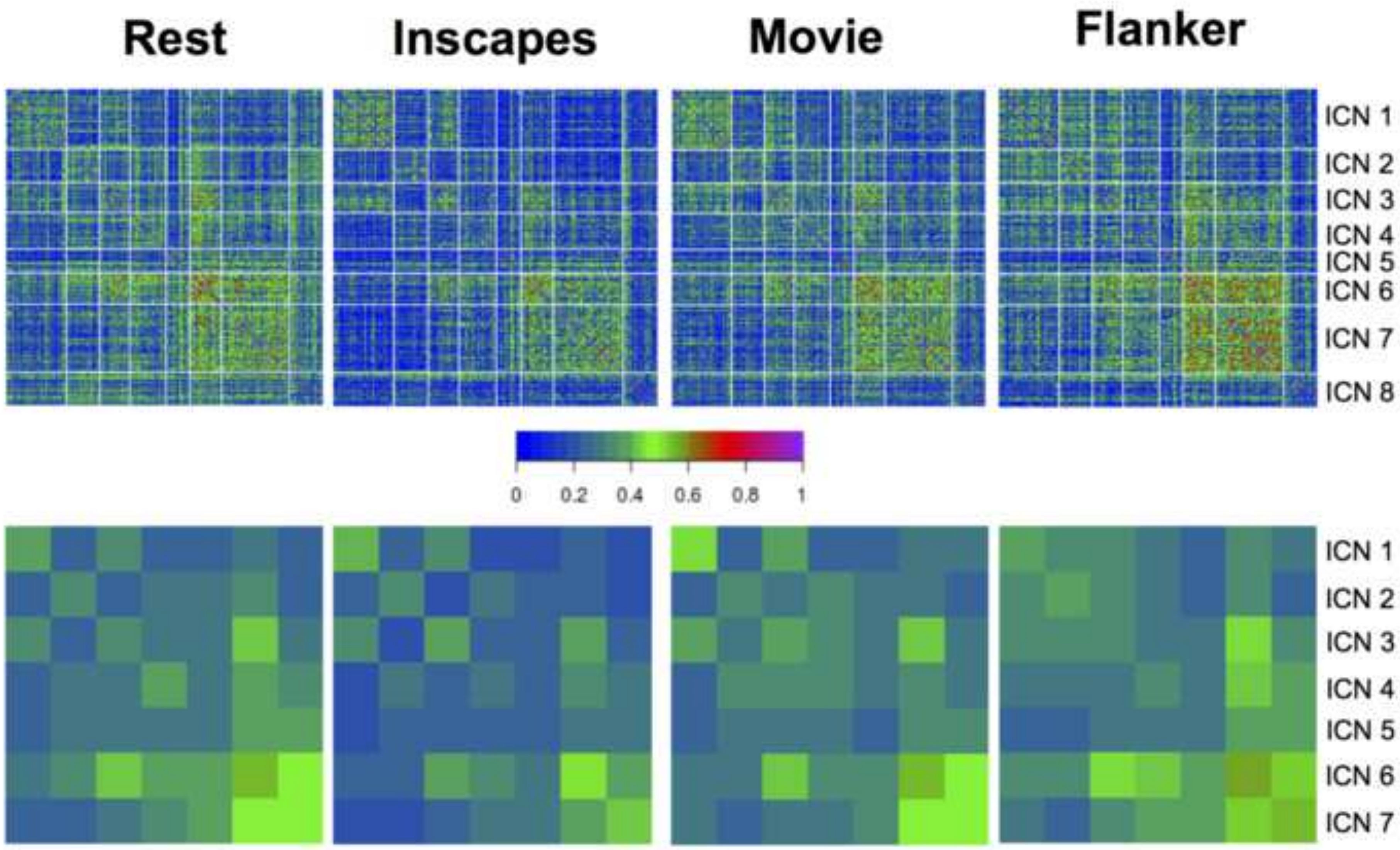

Intrinsic Connectivity Networks

ICN 1 - Visual	ICN 5 - Limbic
ICN 2 - Somatomotor	ICN 6 - FrontoParietal
ICN 3 – Dorsal Attention	ICN 7 – Default
ICN 4 – Ventral Attention	ICN 8 – No Overlap
