# Supplementary material for: The Healthy Brain Network Serial Scanning Initiative: a resource for evaluating inter-individual differences and their reliabilities across scan conditions and sessions
Source: Gigascience. 2017 Jan 7;6(2):1–14. doi: 10.1093/gigascience/giw011 (PMC5466711; doi:10.1093/gigascience/giw011)
Supplement: Response_to_Reviewer_Comments_Original_Submission.pdf [file giw011_Response_to_Reviewer_Comments_Original_Submission.pdf]

Dear Editor,

We appreciated the overall positive feedback regarding our submission, and are pleased to submit a revised version of our manuscript titled "The Healthy Brain Network Serial Scanning Initiative: A resource for evaluating inter-individual differences and their reliabilities across scan conditions and sessions." for publication in GigaScience. We have carefully reviewed the thoughtful feedback provided by the reviewers, and integrated their suggestions. Below we provide a point-by-point response to facilitate review of our changes.

Reviewer 1:
1. Abstract: in findings rather than 'reliability analyses' I suggest naming them or adding a parenthesis and list metrics used

Response: We have modified the sentence in the abstract to read as follows: "Technical validation and demonstrative reliability analyses were carried out at the connection-level using the Intraclass Correlation Coefficient (ICC), and at network level representations of the data using the Image Intraclass Correlation Coefficient (I2C2). Variation in intrinsic functional connectivity across sessions was generally found to be greater than that attributable to scan condition."

2. Description of movie clips used - I think you need to specify the version of the movie - I'm sure my French version of the matrix starting at 00.25.23.10 is different from yours ... also are the clips shared too ? if so please mention it

Response: We have added version information for the movies used to the manuscript and improved our descriptions of Inscapes. Unfortunately the movie clips cannot be directly shared due to copyright issues, however we have shared the Inscapes clips on the Downloads section of the project webpage (http://fcon_1000.projects.nitrc.org/indi/hbn_ssi/download.html).

3. Same for the flanker task - is it shared?

Response: We have now shared the Flanker task stimuli, and their sequences, in the Downloads section of the project webpage (http://fcon_1000.projects.nitrc.org/indi/hbn_ssi/download.html).

4. Data Privacy - please name and reference the software used to defacing

Response: We have specified and cited the defacing software used in the Data Privacy section, as follows: "The removal of facial features as performed using the "Face Masking" software package developed by the Washington University Neuroinformatics Research Group [41]."

5. Data preprocessing - fingerprinting: what did you use for correlation? in the figure 5 it says Pearson - in that case, why did you write 'spatial correlation' ; Cameron likes concordant correlation ratio - it's more spatial -- please specify and maybe include some measures ie mean correlations and 95% CI (I prefer Bayesian CI but it's up to you)

Response: In accord with the reviewer's comments, we have amended the description of the correlation used in the Fingerprinting section, and added in the mean, standard deviation, and 95% confidence intervals for within and between comparisons. The text now reads as follows: "Consistent with their work, we found a dramatically higher degree of correlation, using Pearson's R, between connectivity matrices obtained from the same individual on differing sessions (Mean: 0.599, Standard Deviation: 0.083, 95% Confidence Interval: 0.598 – 0.600), when compared to differing individuals (Mean: 0.445, Standard Deviation: 0.065, 95% Confidence Interval: 0.444 – 0.445) (Figure 5)."

6. Same with connection wise ICC the mean values with 95& CI for between vs within might be useful to

report

Response: With regard to the distribution of values for our ICC based test-retest analysis of the data, we have added Table 5 describing the mean, standard deviation and 95% confidence intervals for within and between network comparisons, for each scan condition. The additional table and caption are provided in the revised manuscript, and the caption is also provided below:

“Table 5 – Displayed here are summary statistics of the distribution of ICC values from the test-retest reliability analysis of each scan condition. Shown are the mean, standard deviation (Std. Dev.), and 95% confidence interval (95% C.I.) of ICC values for within network and between network connections.”

Reviewer 2:
While there is no concern about the methods used to acquire the data or the analysis, there is some concern that a 1.5T magnet was used in this study. Although it was stated that the data was acquired as part of a "pilot initiative being carried out to evaluate the capabilities of a 1.5T mobile scanner when equipped with a state-of-the-art head coil and imaging sequences", the usefulness of the results are tempered by most research of this type being performed on 3.0T scanners. This limits the comparative value of the results in this study

Response: We appreciate the reviewer's concern, as 3T scanners have higher SNR that 1.5T. But, we would also like to emphasize that the system we used has been updated to 32 receive channels to support the latest in head coil technology that offers a substantial improvements in SNR over data collected in previous generations of 1.5T scanners. Also, we utilized simultaneous multi-slice imaging which allowed us to improve the spatial and temporal resolution of the data over what has been typically collected at 1.5T. While we do expect to see some differences between results at 1.5T and 3T, we do not believe that the overall results should fundamentally differ. In response to the reviewer’s concern, we have added the following statement of limitation to the manuscript:

“A limitation of the described resource is that the data was collected using a 1.5T scanner platform, rather than 3T. While we do not expect the overall results obtained with data from the 1.5T and 3T platforms should be fundamentally different, there is generally better SNR and temporal resolution with the 3T scanner platform. To mitigate these differences, 1) the system was upgraded to 32 receive channels to take advantage of the latest head-coil technologies for increasing SNR, and 2) simultaneous multi-slice imaging was used to improve the spatial and temporal resolution. ”

1. Data Description: In "…of the participant during scanning can effect iFC patterns;…"; replace "effect" with "affect"

Response: In the section Data Description, the following line "…of the participant during scanning can effect iFC patterns;…"; was changed to ."…of the participant during scanning can affect iFC patterns;…"; was

2. "Magnetization" rather than "Myelin".

Response: In Table 1, in the baseline characterization section, "Myelin Transfer Ratio" has been changed to "Magnetization Transfer Ratio".

3. "included with the BIDS organized imaging data as tab separate values (TSV) files."; should be "…BIDS-organized…"

Response: On page 12, the line "included with the BIDS organized imaging data as tab separate values (TSV) files."; was changed to "included with the BIDS-organized imaging data as tab separate values (TSV) files.";

4. Figure 2 caption: Since this is an acronym "Entropy focus criterion (EFC)" should be "Entropy Focus

Criterion (EFC)".

Response: In the Figure 2 caption: "Entropy focus criterion (EFC)" was changed to "Entropy Focus Criterion (EFC)".

5. Fig. 2 caption: "the data t across scan conditions". The "t" can be removed as it doesn't show up in the figure and so doesn't contribute to any information transfer at this point.

Response: In the Figure 2 caption, "the data t across scan conditions" was changed to "the data across scan conditions".

6. Fig. 3 caption: Since this is an acronym "Entropy focus criterion (EFC)" should be "Entropy Focus Criterion (EFC)".

Response: In the Figure 3 caption "Entropy focus criterion (EFC)" was changed to "Entropy Focus Criterion (EFC)".

7. Fig. 3 caption: "the data t across scan conditions". The "t" should be removed.

Response: In the Figure 3 caption, "the data t across scan conditions" was changed to "the data across scan conditions".

8. Fig. 4 caption: In "Outliers Detection", "detection" should not be capitalized if it's not going to be used as an acronym "(OD)". "t" can also be removed.

Response: In the Fig. 4 caption, "Outliers Detection" was changed to "Outliers detection", "the data t across scan conditions" was changed to "the data across scan conditions".

9. Table 4's caption should state that these values are ICC values.

Response: The caption for Table 4 was amended to include that the values are ICC values; as show here: "Table 4 – ICC values representing the test-retest reliability of Quality Assurance Protocol (QAP) measures, for each scan condition."

10. The reconstructed resolution for 3D-FLASH is missing the third dimension.

Response: The third dimension of reconstructed resolution from the 3D-FLASH acquisition parameters was added to Table 2.

11. DKI pulse sequence: is that 64 gradient directions for each b-value for 64 directions total?

Response: The DKI pulse sequence does indeed have 64 gradient directions for each b-value, for 64 directions total.

12. The number of b=0 scans for the DKI scan is smaller than is usual for that number of gradient directions. Was there a reason for this?

Response: We had attempted to collect more b=0 scans, but the data ended up corrupted, so we reduced the number.

13. What is the DWI scan? It is listed as having a b-value = 0, but 64 directions?

Response: The DWI scans were intended to be collected as extra b=0 scans for the DKI scan, as well as for

mapping the inhomogeneity in the magnetic field (We collected two DWI images with opposing frequency encoding directions). However the DWI scan parameters needed to be changed significantly from those of the DKI scan in order to correctly map the magnetic field, therefore these will just serve as a tool for performing distortion correction in data preprocessing.

14. It would be clearer in the scan parameter table to say "Number of b=0 scans" as opposed to "Number of B Zeros"

Response: In Table 2 the variable described as "Number of B Zeros" was changed to "Number of b=0 scans.

15. Top of page 17: To help readability the first full sentence should start with "The variables …

Response: The authors are not sure which sentence Reviewer 2 is referring to with this point.

Further to the above amendments, we also noticed a typo in figure 7. The scan condition “Inscapes” was referred to as “Inscap”. This has been fixed.

We hope these responses satisfy the reviewer’s comments and enquiries. We eagerly anticipate your reply.

Yours Sincerely,

Michael P. Milham, MD, PhD

Phyllis Green and Randolph Cowen Scholar
Director, Center for the Developing Brain
Child Mind Institute
http://www.childmind.org/

Director, Center for Biomedical Imaging and Neuromodulation
Research Psychiatrist
Nathan S. Kline Institute for Psychiatric Research
