# Supplementary material for: The Healthy Brain Network Serial Scanning Initiative: a resource for evaluating inter-individual differences and their reliabilities across scan conditions and sessions
Source: Gigascience. 2017 Jan 7;6(2):1–14. doi: 10.1093/gigascience/giw011 (PMC5466711; doi:10.1093/gigascience/giw011)
Supplement: Response_to_Reviewer_Comments_Revision_1.pdf [file giw011_Response_to_Reviewer_Comments_Revision_1.pdf]

Updated manuscript with GigaScience DOI, and correct face masking software.

Updated Ref 71 with DOI citation details.
