# Supplementary material for: The Healthy Brain Network Serial Scanning Initiative: a resource for evaluating inter-individual differences and their reliabilities across scan conditions and sessions
Source: Gigascience. 2017 Jan 7;6(2):1–14. doi: 10.1093/gigascience/giw011 (PMC5466711; doi:10.1093/gigascience/giw011)
Supplement: Reviewer_1_Report_(Original_Submission).pdf [file giw011_Reviewer_1_Report_(Original_Submission).pdf]

Reviewer Report

Title: "The Healthy Brain Network Serial Scanning Initiative: A resource for evaluating inter-individual differences and their reliabilities across scan conditions and sessions"

Version: Original Submission **Date:** 10/13/2016

Reviewer name: Cyril Pernet

Reviewer Comments to Author:

The authors describe an additional (small) data-set available with the 1000 connectome projects ; and in itself it's a nice dataset.

I have not downloaded and reviewed the data - so my comments relates to the manuscript only. These are more suggestion than fundamental disagreement with what is in the manuscript.

(1) Abstract: in findings rather than 'reliability analyses' I suggest naming them or adding a parenthesis and list metrics used

(2) Description of movie clips used - I think you need to specify the version of the movie - I'm sure my French version of the matrix starting at 00.25.23.10 is different from yours ... also are the clips shared too ? if so please mention it

(3) Same for the flanker task - is it shared?

(4) Data Privacy - please name and reference the software used to defacing

(5) Data preprocessing - fingferprinting: what did you use for correlation? in the figure 5 it says Pearson - in that case, why did you write 'spatial correlation' ; Cameron likes concordant correlation ratio - it's more spatial -- please specify and maybe include some measures ie mean correlations and 95% CI (I prefer Bayesian CI but it's up to you)

(6) same with connection wise ICC the mean values with 95& CI for between vs within might be useful to report

Cyril Pernet

Level of Interest

Please indicate how interesting you found the manuscript: An article whose findings are important to those with closely related research interests

Quality of Written English

Please indicate the quality of language in the manuscript: Acceptable

Declaration of Competing Interests

Please complete a declaration of competing interests, considering the following questions:

- Have you in the past five years received reimbursements, fees, funding, or salary from an organisation that may in any way gain or lose financially from the publication of this manuscript, either now or in the future?
- Do you hold any stocks or shares in an organisation that may in any way gain or lose financially from the publication of this manuscript, either now or in the future?
- Do you hold or are you currently applying for any patents relating to the content of the manuscript?
- Have you received reimbursements, fees, funding, or salary from an organization that holds or has applied for patents relating to the content of the manuscript?
- Do you have any other financial competing interests?
- Do you have any non-financial competing interests in relation to this paper?

If you can answer no to all of the above, write 'I declare that I have no competing interests' below. If your reply is yes to any, please give details below.

I declare that I have no competing interests

I agree to the open peer review policy of the journal. I understand that my name will be included on my report to the authors and, if the manuscript is accepted for publication, my named report including any attachments I upload will be posted on the website along with the authors' responses. I agree for my report to be made available under an Open Access Creative Commons CC-BY license (http://creativecommons.org/licenses/by/4.0/). I understand that any comments which I do not wish to be included in my named report can be included as confidential comments to the editors, which will not be published.

I agree to the open peer review policy of the journal
