# Supplementary material for: The Healthy Brain Network Serial Scanning Initiative: a resource for evaluating inter-individual differences and their reliabilities across scan conditions and sessions
Source: Gigascience. 2017 Jan 7;6(2):1–14. doi: 10.1093/gigascience/giw011 (PMC5466711; doi:10.1093/gigascience/giw011)
Supplement: Reviewer_2_Report_(Original_Submission).pdf [file giw011_Reviewer_2_Report_(Original_Submission).pdf]

Reviewer Report

Title: "The Healthy Brain Network Serial Scanning Initiative: A resource for evaluating inter-individual differences and their reliabilities across scan conditions and sessions"

Version: Original Submission **Date:** 10/20/2016

Reviewer name: Karl Helmer

Reviewer Comments to Author:

This manuscript describes the data acquired by, and some sample results from, the Healthy Brain Network Serial Scanning Initiative. The manuscript is well written and the data and sample analyses are clearly described. The description of the data is generally complete and informative.

The study is well designed, especially counterbalancing the different scan conditions. The resulting data set has a wide range of possible uses given the variety of data acquired.

While there is no concern about the methods used to acquire the data or the analysis, there is some concern that a 1.5T magnet was used in this study. Although it was stated that the data was acquired as part of a "pilot initiative being carried out to evaluate the capabilities of a 1.5T mobile scanner when equipped with a state-of-the-art head coil and imaging sequences", the usefulness of the results are tempered by most research of this type being performed on 3.0T scanners. This limits the comparative value of the results in this study.

Specific Comments:

1) Data Description: In "…of the participant during scanning can effect iFC patterns;…"; replace "effect" with "affect"

2) Table 1: "Magnetization" rather than "Myelin".

3) Page 12: "included with the BIDS organized imaging data as tab separate values (TSV) files."; should be "…BIDS-organized…"

4) Figure 2 caption: Since this is an acronym "Entropy focus criterion (EFC)" should be "Entropy Focus Criterion (EFC)".

5) Fig. 2 caption: "the data t across scan conditions". The "t" can be removed as it doesn't show up in the figure and so doesn't contribute to any information transfer at this point.

6) Fig. 3 caption: Since this is an acronym "Entropy focus criterion (EFC)" should be "Entropy Focus Criterion (EFC)".

7) Fig. 3caption: "the data t across scan conditions". The "t" should be removed.

8) Fig. 4 caption: In "Outliers Detection", "detection" should not be capitalized if it's not going to be used as an acronym "(OD)". "t" can also be removed.

9) Table 4's caption should state that these values are ICC values.

10) The reconstructed resolution for 3D-FLASH is missing the third dimension.

11) DKI pulse sequence: is that 64 gradient directions for each b-value for 64 directions total?

12) The number of b=0 scans for the DKI scan is smaller than is usual for that number of gradient directions. Was there a reason for this?

13) What is the DWI scan? It is listed as having a b-value = 0, but 64 directions?

14) It would be clearer in the scan parameter table to say "Number of b=0 scans" as opposed to "Number of B Zeros"

15) Top of page 17: To help readability the first full sentence should start with "The variables …

Level of Interest

Please indicate how interesting you found the manuscript: An article whose findings are important to those with closely related research interests

Quality of Written English

Please indicate the quality of language in the manuscript: Acceptable

Declaration of Competing Interests

Please complete a declaration of competing interests, considering the following questions:

- Have you in the past five years received reimbursements, fees, funding, or salary from an organisation that may in any way gain or lose financially from the publication of this manuscript, either now or in the future?
- Do you hold any stocks or shares in an organisation that may in any way gain or lose financially from the publication of this manuscript, either now or in the future?
- Do you hold or are you currently applying for any patents relating to the content of the manuscript?

- Have you received reimbursements, fees, funding, or salary from an organization that holds or has applied for patents relating to the content of the manuscript?
- Do you have any other financial competing interests?
- Do you have any non-financial competing interests in relation to this paper?

If you can answer no to all of the above, write 'I declare that I have no competing interests' below. If your reply is yes to any, please give details below.

I declare that I have no competing interests.

I agree to the open peer review policy of the journal. I understand that my name will be included on my report to the authors and, if the manuscript is accepted for publication, my named report including any attachments I upload will be posted on the website along with the authors' responses. I agree for my report to be made available under an Open Access Creative Commons CC-BY license (http://creativecommons.org/licenses/by/4.0/). I understand that any comments which I do not wish to be included in my named report can be included as confidential comments to the editors, which will not be published.

I agree to the open peer review policy of the journal
